# A Systematic Literature Review of Neuroimaging of Psychopathic Traits

**DOI:** 10.3389/fpsyt.2019.01027

**Published:** 2020-02-06

**Authors:** Mika Johanson, Olli Vaurio, Jari Tiihonen, Markku Lähteenvuo

**Affiliations:** ^1^ Department of Clinical Neuroscience, Karolinska Institute, Stockholm, Sweden; ^2^ Department of Forensic Psychiatry, Niuvanniemi Hospital, Kuopio, Finland; ^3^ Department of Forensic Psychiatry, University of Eastern Finland, Kuopio, Finland

**Keywords:** psychopathy, neuroimaging, review, antisocial, callous-unemotional, emotional detachment

## Abstract

**Introduction:**

Core psychopathy is characterized by grandiosity, callousness, manipulativeness, and lack of remorse, empathy, and guilt. It is often comorbid with conduct disorder and antisocial personality disorder (ASPD). Psychopathy is present in forensic as well as prison and general populations. In recent years, an increasing amount of neuroimaging studies has been conducted in order to elucidate the obscure neurobiological etiology of psychopathy. The studies have yielded heterogenous results, and no consensus has been reached.

**Aims:**

This study systematically reviewed and qualitatively summarized functional and structural neuroimaging studies conducted on individuals with psychopathic traits. Furthermore, this study aimed to evaluate whether the findings from different MRI modalities could be reconciled from a neuroanatomical perspective.

**Materials and Methods:**

After the search and auditing processes, 118 neuroimaging studies were included in this systematic literature review. The studies consisted of structural, functional, and diffusion tensor MRI studies.

**Results:**

Psychopathy was associated with numerous neuroanatomical abnormalities. Structurally, gray matter anomalies were seen in frontotemporal, cerebellar, limbic, and paralimbic regions. Associated gray matter volume (GMV) reductions were most pronounced particularly in most of the prefrontal cortex, and temporal gyri including the fusiform gyrus. Also decreased GMV of the amygdalae and hippocampi as well the cingulate and insular cortices were associated with psychopathy, as well as abnormal morphology of the hippocampi, amygdala, and nucleus accumbens. Functionally, psychopathy was associated with dysfunction of the default mode network, which was also linked to poor moral judgment as well as deficient metacognitive and introspective abilities. Second, reduced white matter integrity in the uncinate fasciculus and dorsal cingulum were associated with core psychopathy. Third, emotional detachment was associated with dysfunction of the posterior cerebellum, the human mirror neuron system and the Theory of Mind denoting lack of empathy and persistent failure in integrating affective information into cognition.

**Conclusions:**

Structural and functional aberrancies involving the limbic and paralimbic systems including reduced integrity of the uncinate fasciculus appear to be associated with core psychopathic features. Furthermore, this review points towards the idea that ASPD and psychopathy might stem from divergent biological processes.

## Introduction

Psychopathy is linked to biological processes in the brain, and is a highly heritable disorder (1). Structural and functional magnetic resonance imaging (MRI) have provided means to investigate these processes, but both the results and the definition of psychopathy have been heterogenic (2–4). Features and behaviors, such as lack of empathy, remorse, and guilt as well as manipulativeness, callousness, and grandiosity comprise the core psychopathic traits. Antisocial conduct is often comorbid with these core traits, which together are referred as to psychopathy (5–7).

The display of psychopathic behaviors is a reliable predictor for poor academic achievement, criminality, behavioral problems, and for adverse psychosocial consequences and mental health (8, 9). The prevalence of psychopathy is approximately 1% in the general population (10, 11), 3% in forensic population (12), 4% amongst corporate managers (13), and 20% in prison population (14). Furthermore, conduct disorder (CD) is often present amongst the majority of offenders with clinical psychopathy before the age of fifteen, and antisocial personality disorder (ASPD) after the age of eighteen (15). The PCL-R superordinate interpersonal-affective factor of psychopathy is not a prerequisite for CD and ASPD, but they are, however, often comorbid (16). Moreover, psychopaths having successfully avoided criminal conviction are sometimes referred to as successful psychopaths (17). However, in this context, the word “successful” does not imply success in other aspects of life (17).

Psychopathy is believed to have a neurobiological origin (18), and, in the past years, various neuroimaging studies have tried to resolve the perplexing etiology behind psychopathy (2, 4, 19). The structure, connectivity, and white matter tracts of brains of individuals displaying psychopathic traits have been visualized with numerous methods including conventional MRI, functional MRI, diffusion tensor MRI (DTI) voxel-based morphometry (VBM), (19), single photon emission computed tomography (SPECT), positron emission tomography (PET), and electroencephalogram (EEG) (20).

Despite an increase in neuroimaging studies in this field, there is no systematic review summarizing structural MRI, functional MRI, and DTI findings to date. Previous reviews have yielded inconsistent results [see e.g. (2, 3, 18)]. Diversity in sample demographics and characteristics as well as variation in task designs and imaging techniques make the interpretation and generalization of neuroimaging results difficult (3). Put differently, the functions, structures, and interconnections of brain regions associated with psychopathy remain unclear. A qualitative summary covering the three radiological submodalities might facilitate our understanding of psychopathy, and give insight to its neurobiological correlates and obscure neurobiological etiology.

## Aim

The aim of this study was to conduct a systematic literature review on MRI neuroimaging of psychopathic traits, to summarize findings from different MRI modalities that cover different aspects of neural function and structure, and to examine whether these aspects were consistent.

## Materials and Methods

### Study Design

This study is a systematic literature review on MRI neuroimaging of psychopathic traits, conducted per the Preferred Reporting Items for Systematic Reviews and Meta-Analyses (PRISMA) Statement (21).

### Inclusion and Exclusion Criteria

For inclusion in the study, the record must have been published in a peer-reviewed journal in English, Finnish or Swedish. Because psychopathy is prevalent in various populations and both genders, we also included community samples in addition to prison and forensic populations. Consequently, both genders were included in our study. Furthermore, most records use PCL-R (5–7) as the measure of psychopathy, but records with PCL-R derived instruments were also included.

If the sample mean age was less than 17.50 years, the record was excluded. This criterion applied to both affected subjects and control groups. This criterion resulted in exclusion of early adolescence studies, but allowed room for late adolescence studies.

### Data Collection

The following databases were accessed to acquire records for study: PubMed (NCBI), Medline (Ovid), PsycINFO (Ovid), PsycARTICLES (Ovid), Embase, and Criminal Justice Abstracts (EBSCO). The search was executed on the 4^th^ of February 2019. Apart from categorical psychopathy, search strings, such as callous-unemotional traits, conduct disorder, and antisocial behavior, were used in order to encapsulate the dimensional continuum of psychopathy. The search strings and methods are available in **Supplementary Materials**.

**Figure 1:**
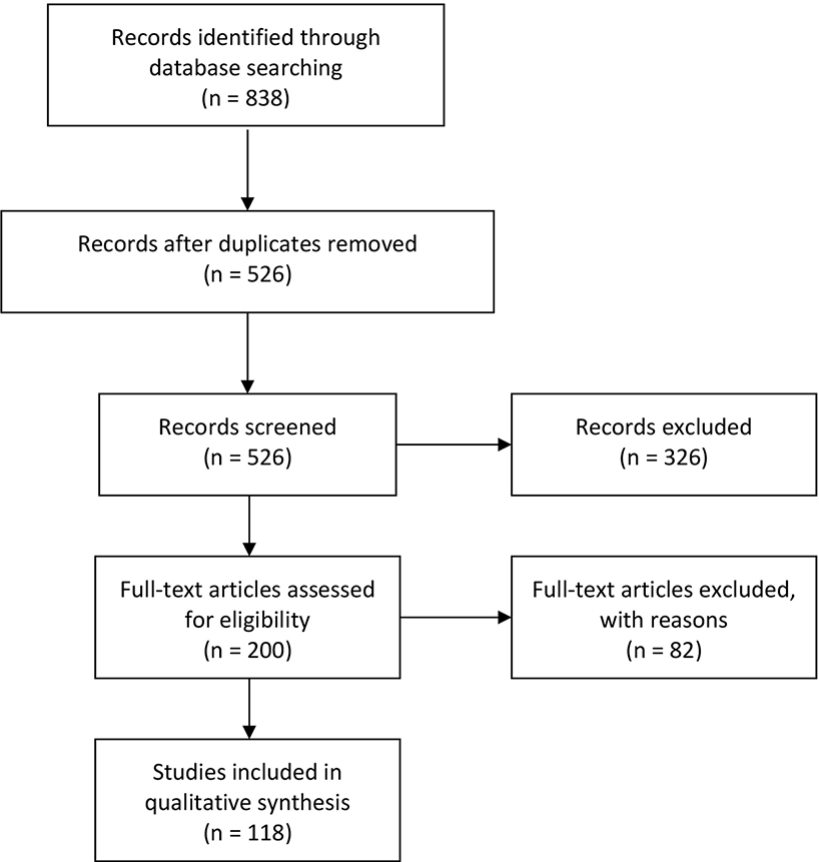
A flowchart of the screening process.

The screened records (n = 526) were rated for either inclusion or exclusion by three independent assessors at the Niuvanniemi Hospital (BM Mika Johanson and forensic psychiatrics MD Olli Vaurio, and MD, PhD Markku Lähteenvuo). The initial interrater reliability for inclusion and exclusion was estimated with Fleiss' Kappa, reaching a Kappa value of 0.942 and an initial agreement percent across the raters of 97.34%. All articles with initial disagreement were re-rated within the group, and a decision for either inclusion or exclusion was made in consensus. As a result, a total of 118 records were included in the study and 408 excluded (**Figure 1**). The characteristics and key findings of each included study are summarized in the Review Matrix (**Table S1**). Excluded records with reasons for exclusion are available in **Table S2**.

### Coding and Analysis

Data from the included records (n = 118) were extracted and coded to form the review matrix (**Table S1**). The coded data included author and year of the record, type, and design of the study, sample characteristics, exclusion criteria, covariates, behavioral measures, MRI modality and method, and key findings. Every record was assigned with a unique and corresponding number. Type and design of the study included also the mean psychopathy score for the sample. Sample characteristics included sample size, mean age, and percentage of females. Based on the data in the review, matrix, functional, structural, and diffusion tensor MRI findings that correlated with psychopathy dimensionally or categorically, were compiled to **Table 1**. Findings that correlated with core psychopathy only were compiled to **Table 2**.

**Table 1:** Key neuroanatomical areas affected in psychopathy categorically and dimensionally. The records are grouped by method. Dimensional correlations in terms of total psychopathy score are shown.

No	Record	Method	AMY	HIP	INS	CG	PFC	TEMP	FUSI	PAR	OCC	CAU	PUT	CB	VS	WMT
**52**	Baskin-Sommers et al. (22)	S	g-	g-												
**17**	Bertsch et al. (23)	S				↓g	↓g			↓g	↓g					
**1**	Boccardi et al. (24)	S		m ↓g-												
**22**	Boccardi et al. (25)	S	m ↑g		↓g	↓g	↓g		↓g	↓g	↓g					
**11**	Boccardi et al. (26)	S										m	m		↓g m	
**40**	Contreras-Rodríguez et al. (27)	S	↓g	↓g	↓g	↓g	↓g	↓g	↓g	↓g						
**38**	Cope et al. (28)	S		g-	g-	g+	g+	g-			g-	g+				
**85**	de Oliveira-Souza et al. (29)	S			↓g	g-	↓g-	↓g-								
**51**	Ermer et al. (30)	S	↓g-	↓g-		↓g-	↓g-	↓g-								
**16**	Fairchild et al. (31)	S	↓g		↓g		↓g	↑g	↓g		↓g	↓g				
**48**	Glenn et al. (32)	S										↑g+	↑g+			
**95**	Gregory et al. (33)	S			↓g		↓g	↓g		↓g						
**105**	Howner et al. (34)	S						↓g-								
**45**	Korponay et al. (35)	S											↑g+		↑g+	
**46**	Korponay et al. (36)	S					↑g+									
**75**	Laakso et al. (37)	S					↓g									
**12**	Leutgeb et al. (38)	S					↓g					↑g		↑g		
**24**	Ly et al. (39)	S			↓g	↓g	↓g	↓g	↓g		↓g					
**53**	Miskovich et al. (40)	S				m-	m-			m-						
**41**	Müller et al. (41)	S				↓g	↓g	↓g								
**60**	Pardini et al. (42)	S	↓g-													
**21**	Raine et al. (43)	S														↑w CR, CC
**42**	Raine et al. (44)	S		m												
**43**	Sato et al. (45)	S				↓g-		↓g-			↓g-					
**13**	Tiihonen et al. (46)	S		↓g	↓g	↓g	↓g	↓g		↓g ↑w	↑w			↑g ↑w		
**78**	Vieira et al. (47)	S	↓g-				↑g+					↑g+	↓g-			
**59**	Yang et al. (48)	S	↓g													
**62**	Yang et al. (17)	S	↓g m			↓g	↓g	↓g								
**28**	Anderson et al. (49)	F			a+		a+				a+/−					
**76**	Bjork et al. (50)	F													a+	
**30**	Contreras-Rodríguez et al. (51)	F	↓a ↓c				↑a ↓c		↓c	↓c	↑a ↓c					
**40**	Contreras-Rodríguez et al. (27)	F	↓c		↓c		↑c ↓c									
**83**	Cope et al. (52)	F	↑a	↑a	↑a	↑a−	a−			a−		↑a	↑a−		↑a	
**6**	Decety et al. (53)	F			↑a+/− ↑↓c	↑a ↓c	↑a+/− ↓c	↑a ↓c				↑a				
**14**	Decety et al. (54)	F			↑a+	↑a−	↑a+/−	↑a+/−		↑a+/−						
**71**	Decety et al. (55)	F	↑a		↑a+	↑a−	a−	↑a−	a−	↑a−	a−					
**39**	Deeley et al. (56)	F			↑a	↑a	↑a		↑a	↑a	↑a			↑a		
**82**	Deming et al. (57)	F				↑a	↑↓a	↑a		↑↓a						
**81**	Ewbank et al. (58)	F	↓a c−			c−										
**33**	Fede et al. (59)	F				a−	a−									
**63**	Geurts et al. (60)	F					↑c								↑a ↑c	
**9**	Glenn et al. (61)	F			a+	a+/−	a+/−			a+/−						
**87**	Gregory et al. (62)	F				↑a+		↓a		↑a						
**56**	Harenski et al. (63)	F	a−				a−	↑a								
**64**	Harenski et al. (64)	F		a−					av							
**31**	Hosking et al. (65)	F					cv								c− a+	
**4**	Hyde et al. (66)	F	a−													
**50**	Juárez et al. (67)	F		c−		c+/−	c+/−	c+/−		c+/−	c−					
**58**	Kiehl et al. (68)	F	↓a	↓a		↓a	↓a	↓a							↓a	
**45**	Korponay et al. (35)	F									c−		c−			
**96**	Larson et al. (69)	F	↓a				↑a									
**10**	Lindner et al. (70)	F					c+	c+		c+		c+				
**99**	Marsh & Cardinale (71)	F	a−	a−			a+	a−								
**57**	Mier et al. (72)	F	↓c					↓c	↓a							
**89**	Motzkin et al. (73)	F	↓a				↓a			↓a						
**55**	Müller et al. (74)	F	↑a	↓a		↑↓a	↑↓a	↑↓a	↑↓a	↑↓a	↑↓a			↑a		
**3**	Osumi et al. (75)	F	c− a−		c−			a− c−		a−			c− a−		c−	
**15**	Pera-Guardiola et al. (76)	F			↓g											
**54**	Philippi et al. (77)	F			↓c−	↓c−				↓c−						
**66**	Pujara et al. (78)	F													a+/− g+	
**18**	Pujol et al. (19)	F		↓a		↓a− ↓c	↓a ↓c			a−				↓a		
**67**	Rilling et al. (79)	F	↓a−				a+/−									
**8**	Shao and Lee (80)	F				↓a	↓a							↓a		
**47**	Sommer et al. (81)	F					↑a	↑a		↑a						
**32**	Vieira et al. (82)	F				↑a	↑a									
**70**	Yoder et al. (83)	F	↓a− ↓c−	↓a−	↑a+	↑a+ ↑↓c+/−	↑↓a+/− ↓c−	↑a+ ↓c−	↓a−	↓a−	↓a−	↓a− ↓c−	↑a+ ↓c−	↓a−		
**65**	Zijlmans et al. (84)	F					a+	a+								
**102**	Hoppenbrouwers et al. (85)	DTI														↓FA UF ↓FA IFOF ↓FA ATR ↓FA CG
**89**	Motzkin et al. (73)	DTI														↓FA UF
**37**	Sethi et al. (86)	DTI														↓FA CG-
**84**	Sobhani et al. (87)	DTI														↓FA UF-
**103**	Sundram et al. (88)	DTI														↓FA & ↑MD CC ↓FA IC ↓FA & ↑MD IFOF ↓FA & ↑MD ACR ↓FA & ↑MD UF ↓FA ILF ↓FA PTR
**49**	Wolf et al. (89)	DTI														↓FA UF-

AMY, amygdala; HIP, hippocampus including parahippocampal gyri; INS, insula; CG, cingulate gyrus including cingulate cortex; PFC, prefrontal cortex; TEMP, temporal cortex; FUSI, fusiform gyrus; PAR, parietal cortex; OCC, occipital cortex; PUT, putamen; CAU, caudate; CB, cerebellum; VS, ventral striatum including nucleus accumbens; WMT, white matter tract; ATR, anterior thalamic radiation; CC, corpus callosum; CG, cingulum; CR, corona radiata; IC, internal capsule; IFOF, inferior fronto-occipital fasciculus; ILF, inferior longitudinal fasciculus; PTR, posterior thalamic radiation; UF, uncinate fasciculus; S, structural; F, functional; DTI, diffusion tensor imaging; up-and down-arrow denote increase or decrease, respectively; c, functional connectivity; a, activity; g, gray matter volume; w, white matter volume; plus and minus signs denote direction of relationship with psychopathy.

**Table 2:** Key neuroanatomical regions and their correlation to interpersonal-affective dimensions of psychopathy only.

No	Record	Method	AMY	HIP	INS	ACC	PFC	TEMP	CB	DS	WMT
52	Baskin-Sommers et al. (22)	S	g-	g-							
93	Cohn et al. (90)	S	g-		g-						
40	Contreras-Rodríguez et al. (27)	S	g-		g-		g-	g-	g-		
38	Cope et al. (28)	S					g+	g-	g-	g+	
85	de Oliveira-Souza et al. (29)	S				g-	g-	g-			
16	Fairchild et al. (31)	S								g-	
48	Glenn et al. (32)	S								g+	
105	Howner et al. (34)	S						g-			
45	Korponay et al. (35)	S								g+	
86	Lam et al. (91)	S					g-			g-	
12	Leutgeb et al. (38)	S					g-		g+		
53	Miskovich et al. (40)	S				m-					
60	Pardini et al. (42)	S	g-								
78	Vieira et al. (43)	S	g-				g+				
59	Yang et al. (48)	S	g-								
28	Anderson et al. (49)	F			a+		a+				
79	Anderson et al. (92)	F	a−	a−	a−	a−	a−	a−	a−		
27	Cohn et al. (93)	F					c+				
30	Contreras-Rodríguez et al. (51)	F					a+				
40	Contreras-Rodríguez et al. (27)	F					c+				
83	Cope et al. (52)	F			a+		a+		a+	a+	
6	Decety et al. (53)	F	a−		a−		a+/−			a+	
14	Decety et al. (54)	F			a+	a+	a+/−	a+/−			
71	Decety et al. (55)	F			a+		a+/−	a−			
97	Freeman et al. (94)	F				a+					
77	Fullam et al. (95)	F						av			
9	Glenn et al. (61)	F			a+		a+/−				
56	Harenski et al. (63)	F	a−								
64	Harenski et al. (64)	F		a−							
31	Hosking et al. (65)	F					c−				
10	Lindner et al. (16)	F		c+	c+						
94	Murray et al. (96)	F					a−				
54	Philippi et al. (77)	F					c−				
69	Schiffer et al. (97)	F				a−					
72	Seara-Cardoso et al. (98)	F			a−						
7	Seara-Cardoso et al. (99)	F			a−						
35	Vieira et al. (100)	F	a−								
5	Yoder et al. (101)	F	c−			c−	a−		a−		
70	Yoder et al. (83)	F		a−	a+	a+	a−	a+			
65	Zijlmans et al. (84)	F				a−	a−				
102	Hoppenbrouwers et al. (85)	DTI									FA UF- FA IFOF- FA ATR-
80	Pape et al. (102)	DTI									AD CT+
37	Sethi et al. (86)	DTI									FA CG-
49	Wolf et al. (89)	DTI									FA UF-

AMY, amygdala; HIP, hippocampus including parahippocampal gyri; INS, insula; ACC, anterior cingulate cortex (including parts of middle and posterior cingulate cortex); PFC, prefrontal cortex; TEMP, temporal cortex including fusiform gyrus; CB, cerebellum; DS, dorsal striatum; WMT, white matter tract; ATR, anterior thalamic radiation; CG, cingulum; CT, corticospinal tract; IFOF, inferior fronto-occipital fasciculus; UF, uncinate fasciculus; S, structural; F, functional; DTI, diffusion tensor imaging; c, functional connectivity; a, activity; g, gray matter volume; plus and minus signs denote direction of relationship with core psychopathy.

**Figure 2:**
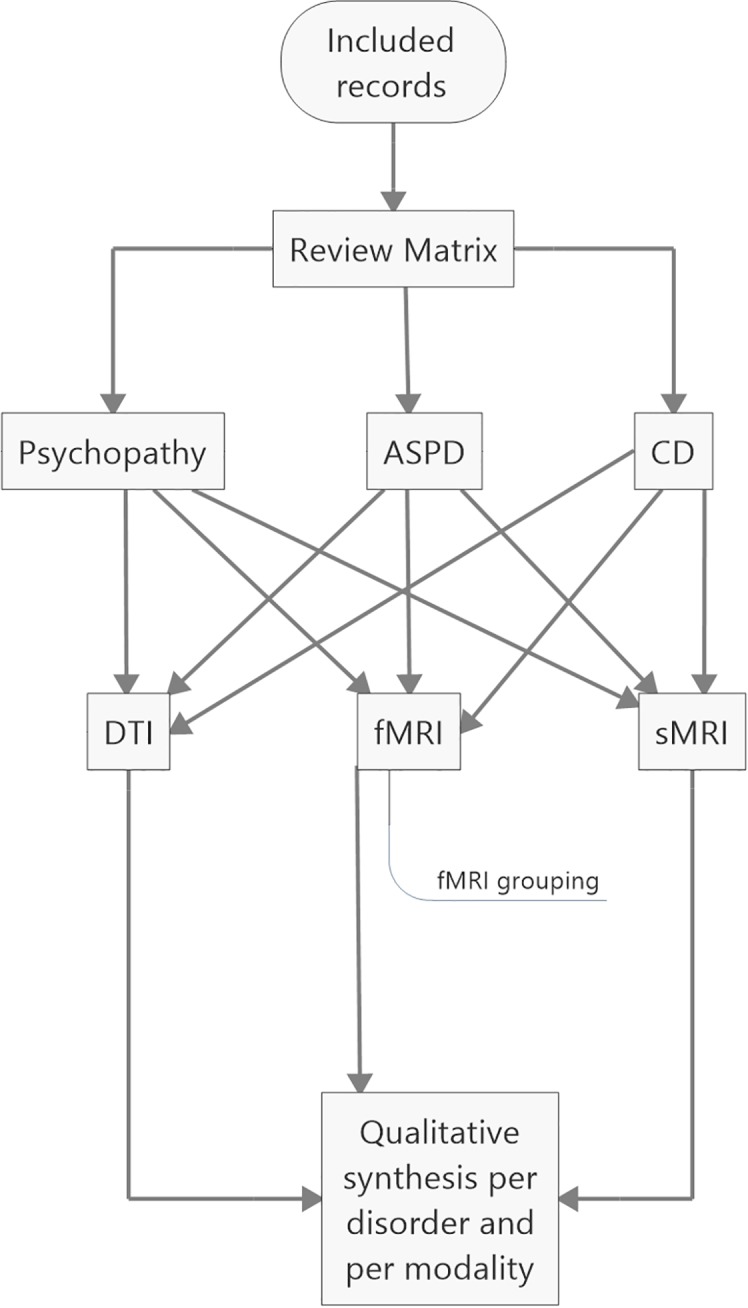
Coding and analyzing processes. ASPD, antisocial personality disorder; CD, conduct disorders; DTI, diffusion tensor MRI; fMRI, functional MRI; sMRI, structural MRI.

Apart from the review matrix, the records were divided into three groups based on whether they aimed to investigate the neural correlates of (i) psychopathy or psychopathic traits, (ii) ASPD, or (iii) CD. The included studies were further divided into structural, functional, and diffusion tensor MRI studies (**Figure 2**). Due to the great number of functional neuroimaging studies of psychopathy, these studies were grouped according to task or setting into six groups (**Table S3**), in order to simplify the summarization process. The six groups were (i) fairness, (ii) moral issue, (iii) viewing affective content, (iv) reward, (v) lying and deception, and (vi) default mode network. The default mode network refers to interconnected areas in the brain, the activity of which reduces in goal-oriented tasks. The areas comprise of ventro- and dorsomedial prefrontal cortex, posterior cingulate cortex, precuneus, and lateral parietal cortex (103). Normal function of the default mode network is associated with self-referential (104), affective (105), and moral cognitive abilities (106, 107).

## Results

**Figure 3:**
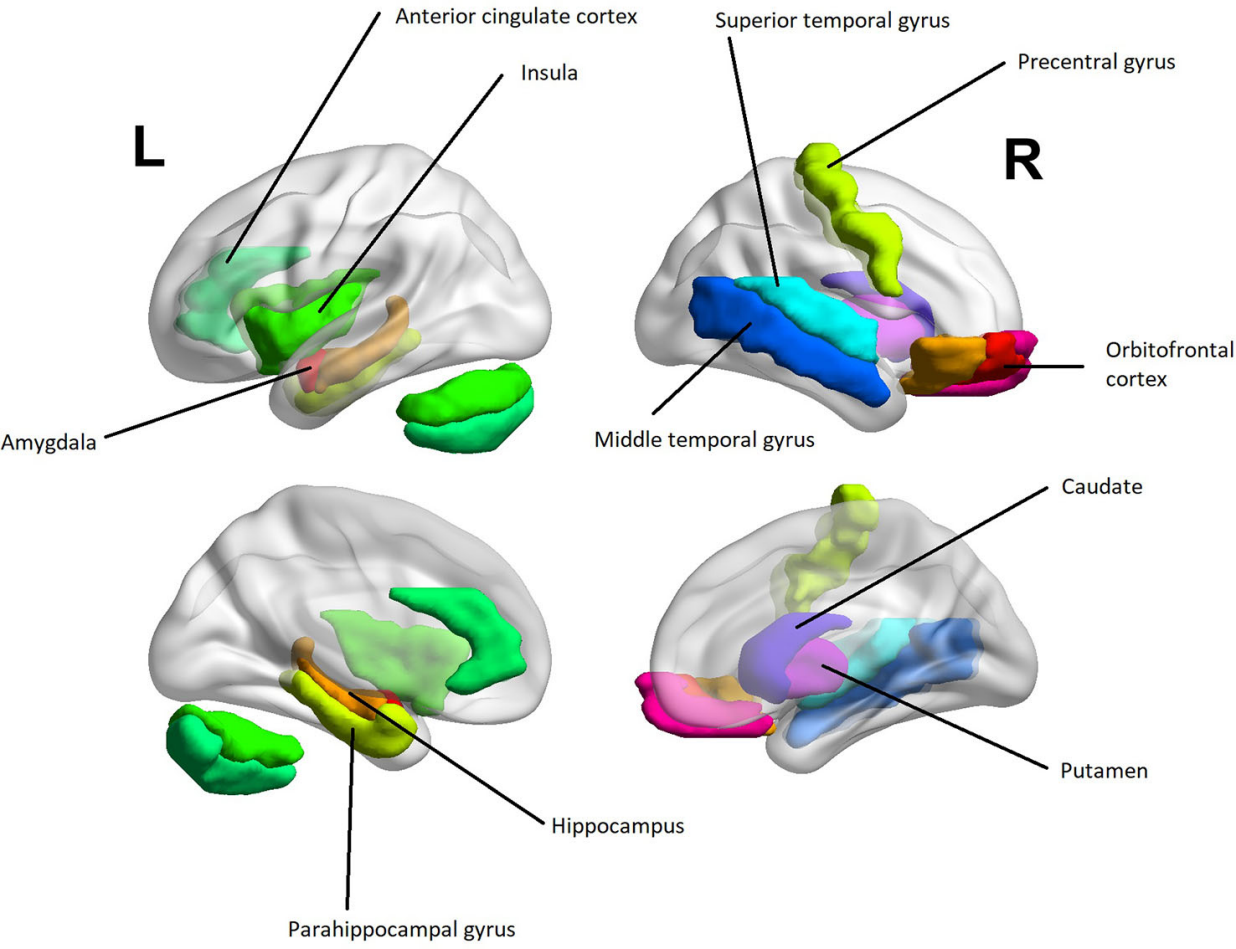
A heuristic anatomical map of brain regions correlating with interpersonal-affective dimensions of psychopathy with lateral and medial views (**Table 2**). The purpose of the figure is to provide an insight into anatomical localizations. To preserve readability, some of the regions are omitted or are present only on the other hemisphere. The visualization was done with the BrainNet Viewer [(107) http://www.nitrc.org/projects/bnv/]. The regions of interest were obtained from the Automated Anatomic Labeling Atlas (108).

Several aberrancies were reported in the psychopathic brain in structural, functional, and diffusion tensor imaging studies. The neuroanatomical regions with most reported aberrancies in individuals with psychopathic traits categorically or dimensionally as a function of total psychopathy score are summarized in **Table 1**. Further, findings correlating with core psychopathy, i.e. interpersonal-affective dimensions only, are summarized in **Table 2**. These areas comprised to great extent of frontotemporal and limbic regions. These areas are also illustrated in **Figure 3**. The prefrontal correlates marked in **Table 2** are divided into functional and anatomical subregions in **Table S4**.

Findings regarding psychopathy are presented first in order of modality. Thereafter, findings related to ASPD and CD are presented and compared to those of psychopathy.

### Structural Gray Matter Findings in Psychopathy

Structurally, aberrancies were described mostly in terms of gray matter volume (GMV) reductions. For a brief summary of implicated brain regions, please see **Table S5**. Moreover and intriguingly, “successful psychopaths” did not show any significant GMV loss compared to healthy controls, whereas their “unsuccessful” counterparts showed prominent losses (17).

#### Prefrontal Cortex

Decreased GMV was reported in several areas of the prefrontal cortex: orbitofrontal cortex (17, 25, 29, 30, 37, 46), dorsomedial prefrontal cortex (23, 33, 38, 39), frontal gyri (25, 33, 37, 39, 41), frontopolar cortex (25, 29, 46), precentral gyri supplementary motor area, sensory motor cortex (25), ventromedial prefrontal cortex, lateral prefrontal cortex (27), and dorsolateral prefrontal cortex (37). However, a few studies reported a positive association between orbitofrontal cortex GMV and degree of psychopathy (28, 36, 47).

#### Temporal Cortex

Decreases in GMV were seen in the temporal regions (17, 110). Most prominent areas of decreased GMV were the superior temporal gyrus (39, 41), middle temporal gyrus (27, 46), superior temporal sulcus (29, 45), fusiform gyrus (25, 27), and the temporal poles (30, 33, 39).

#### Parietal Cortex

A decrease in GMV in the parietal cortices were reported in two notable areas: the precuneus (23, 25, 27) and the postcentral gyrus (23, 33, 46). Moreover, increased white matter volume (WMV) was observed in the occipital and parietal lobes as well as in the left cerebellum (46).

#### Occipital Cortex

The reported GMV reductions in the occipital cortex appeared to be of general nature (23, 39). Areas that were specified include the cuneus (25) and peristriate cortex (45) of the visual processing areas.

#### Limbic Structures

Several regions of the limbic system, the orbitofrontal cortex included (111), showed decreased GMV or abnormal morphology in psychopathy. In particular, the PCL-R superordinate psychopathy was related with decreased GMV across the paralimbic and limbic regions (22).

The amygdalae showed decreased GMV in psychopathy (17, 30, 42, 47, 48, 51). Somewhat contradictory to these findings, Boccardi and colleagues (25) reported larger global amygdalar volumes in a group of psychopathic subjects compared to healthy control group. Further, the amygdalae of psychopathic subjects showed aberrant morphology in the basolateral nuclei (17, 25).

In addition to the amygdalae, the hippocampi (24, 27, 30, 44) and the parahippocampal gyri (30, 46) showed reduced GMV. Further, two studies reported abnormal morphologies in the hippocampi. First, Boccardi and colleagues (24) found that the hippocampi of psychopathic individuals had a double convex morphology in comparison to the normal single convex form. Secondly, Raine et al. (44) found that unsuccessful psychopathic individuals had a volumetric asymmetry in the anterior hippocampi with the right side being larger than the left compared to both successful psychopathic individuals and healthy controls.

Decreased GMV was reported in the subdivisions of the cingulate cortex including the anterior cingulate cortex (25, 39), middle cingulate cortex (41) and posterior cingulate cortex (23, 27, 30, 45, 46). Moreover, psychopathy was associated with abnormal gyrification of the middle cingulate cortex extending into the dorsomedial prefrontal cortex and right parietal cortex in a study by Miskovich and colleagues (40). With respect to the anterior cingulate cortex, divergent results were reported by Glenn, Yang, Raine, and Colletti (112) who did not find differences in volumes between psychopathic individuals and controls. Of note, the control group in this study had a PCL-R mean score of 11.5. Furthermore, a positive correlation between the anterior cingulate cortex volume and psychopathic traits was reported by Cope and colleagues (28).

Also, the insular cortex showed reduced GMV in psychopathy (27, 29, 33, 39, 46, 76).

#### Basal Ganglia

Psychopathy may be accompanied by increased total striatum volume (32, 35). Glenn, Raine, Yaralian, and Yang (32) noted an increase in GMV bilaterally in the globus pallidus, putamen, and in the right caudate body. Similarly, Leutgeb and colleagues (38) showed increased GMV in the left globus pallidus and caudate. The enlarged striatum has also been attributed to bilateral nucleus accumbens and putamen (35). Converging evidence was provided by a positive correlation between GMV in the nucleus accumbens (78), putamen, and caudate (28), and the degree of psychopathy. There are, however, also contradictory results. Firstly, Vieira et al. (47) found increased GMV in the left caudate, but decreased GMV in the left putamen. Secondly, Boccardi et al. (26) did not find any differences in putamen and caudate volumes in psychopathy, albeit the structures manifested aberrant morphology. Moreover, the nucleus accumbens showed a considerable 13% GMV reduction and abnormal morphology (26).

#### Cerebellum

Increased GMV (38, 46) and a positive association between lifestyle-antisocial dimensions of the PCL-R (27) were reported with respect to the cerebellum (38, 46). However, negative associations between cerebellar GMV and interpersonal traits (28) and interpersonal-affective (27) dimensions were also found. Furthermore, decreased cerebellar WMV correlated with psychopathy (113), providing contradictory results to findings mentioned above.

### Functional MRI Findings in Psychopathy

#### Fairness

In functional MRI studies with game-related tasks, psychopathic subjects exhibited reduced amygdalar activity in unfair versus fair conditions (75, 79, 82). Psychopathic subjects showed decreased amygdalar activity when rejecting an unfair offer, and decreased connectivity between amygdala and the limbic regions (75). Osumi and colleagues (75) argue further that amygdalar hypoactivity is indicative of attenuated reactive aggression, allowing the psychopathic subjects to adapt their behavior in order to pursuit personal gain. Furthermore, Viera and colleagues (82) noted that psychopathic subjects showed increased activity in the ventromedial prefrontal cortex and right rostral anterior cingulate cortex in response to unfair offers, whereas the control group showed increased activity in the left dorsolateral prefrontal cortex, which according to Viera et al. (82) implies divergent neural circuitries in decision making.

#### Morality

Several studies implicated dysfunction of the limbic system in psychopathy in the context of moral evaluations (59, 63, 99, 101, 114). Activity in the anterior insular cortex, which modulates anticipated guilt, was attenuated in psychopathic subjects, and the activity negatively correlated with interpersonal psychopathic traits (98). Psychopathic subjects also showed diminished functional connectivity in regions associated with empathetic and emotional processing, specifically between the anterior insular cortex and right temporoparietal junction as well as between the ventromedial prefrontal cortex and amygdala (101). Moreover, hypoactivity in the dorsolateral prefrontal cortex was shown (100). In a similar vein, Pujol et al. (114) found attenuated functional connectivity within the default mode network, particularly between the posterior cingulate cortex and nearby visual areas and medial prefrontal cortex, extending to ventrolateral prefrontal cortex and dorsolateral prefrontal cortex. Psychopathic subjects also demonstrated decreased activity in the hippocampi, posterior cingulate cortex, and medial prefrontal cortex (114). Consistently, Marsh and Cardinale (71) found decreased activity in the right amygdala, parahippocampal gyrus, and precunei.

Moral severity ratings were correlated with increased activity in the right posterior temporal cortex in psychopathic subjects, whereas in the control group ratings were associated with increased activity in the amygdala (63). Furthermore, the control group showed increased activity in the ventromedial prefrontal cortex and anterior temporal cortex during neutral and moral versus non-moral picture recognition (63). This setting was replicated in a female sample, and the results were mostly in line with those of the male sample with temporoparietal hypoactivity being more pronounced in female psychopathic individuals (64). Further, attenuated activity in the posterior cingulate cortex and temporoparietal junction were also seen in psychopathic subjects when judging traits of self and others (57).

#### Default Mode Network

In a great number of studies, the focus lay on investigating connectivity changes of the default mode network. The studies argued further that dysfunction of the default mode network is a key element in psychopathy (27, 67, 70, 73, 77, 92–94, 115). Firstly, decreased functional connectivity was shown between medial-dorsal frontal cortices and limbic regions including the amygdala (27, 73), posterior cingulate cortex (67, 73, 77), insula, and hypothalamus (27). Further supporting limbic and paralimbic dysfunction, Anderson, Maurer, Steele, and Kiehl (92) discovered that core psychopathy was associated with reduced activity in the dorsal anterior cingulate cortex (dACC), posterior cingulate cortex, amygdalae, temporoparietal junction, insula, and parahippocampal gyri, thus also indicative of a dysfunctional salience network. Furthermore, psychopathy was associated with decreased connectivity between the posterior cingulate cortex and parietal cortex (77). Secondly, the medial prefrontal cortex, a subregion of the default mode network, failed to attenuate below baseline in psychopathic subjects at task (94). Somewhat contrariwise, a positive correlation between psychopathic traits and mPFC attenuation was found by Sheng and colleagues (115) in a non-categorial community sample. The researchers did not, however, report the mean or total psychopathy score for the non-categorical sample, leaving the interpretation of the result difficult. Providing further evidence with respect to prefrontal connectivity bias in psychopathy, psychopathic traits were associated with increased functional connectivity between the dorsolateral prefrontal cortex and the medial-dorsal frontal cortices (27), increased connectivity in the frontopolar cortex within the default mode network (93) and more generally in the PFC (36). Thirdly, the correlation between dysfunctional default mode network and psychopathic traits was recently reported in females alike (70).

#### Lying and Deception

Psychopathic subjects showed increased performance in deception and lying (61, 80, 95). Particularly, lying related reductions in activity were seen in the dorsolateral prefrontal cortex, suggestive of prior cognitive training (61, 80). Fullam, McKie, and Dolan (95) did not find activity changes in the dorsolateral prefrontal cortex, but rather increased activity in the ventrolateral prefrontal cortex in all groups. The researchers did, however, conclude similarly that deception is prominent in psychopathy, and it engages more executive cognitive regions of the brain (95).

#### Emotional Detachment

Psychopathic subjects showed decreased ability to recognize and process emotions (51, 72, 74, 76). From a structural viewpoint, emotion recognition was ascribed to the dorsomedial prefrontal cortex, orbitofrontal cortex, anterior insular cortex, and posterior cerebellum in psychopathic subjects, whereas this was attributed to the temporal cortex and amygdala in the control group (76). Functionally, increased activity in the medial prefrontal cortex and visual cortices were seen in psychopathic subjects in emotion recognition, whereas increased amygdalar activity was seen in healthy controls (51). In a similar vein, Volman et al. (116) found decreased functional connectivity between the prefrontal cortex and amygdala in psychopathic subjects in a facial emotion recognition task. Moreover, psychopathic subjects exhibited decreased functional connectivity between bilateral visual prefrontal cortices and the left amygdala, indicative of persistent failure in incorporating emotion into cognition (51).

Somewhat divergent from these findings, firstly, PCL-R score positively correlated with success rate in identifying certain emotions in a study by Decety, Chen, Harenski, and Kiehl (117). Secondly, Anderson et al. (49) found that interpersonal-affective traits correlated with decreased activity in visual cortices. Nonetheless, the researchers did concur with respect to increased activity in the medial prefrontal cortex. Thirdly, no groupwise differences regarding amygdalar activity was found by Deeley and colleagues (56), but they discovered reduced activity in the fusiform gyrus in facial processing in psychopathic subjects. Decety and colleagues (55) discovered similar findings with respect to activity in the amygdala and fusiform gyrus, but noted additionally decreased activity in other areas associated with facial processing, that is, in the superior temporal sulcus, orbitofrontal cortex, inferior occipital gyrus, inferior frontal gyrus (IFG), and ventromedial prefrontal cortex across all emotion ranges. Furthermore, psychopathic subjects showed an increase in activity in the anterior insular cortex in this setting (55). Thirdly, Müller et al. (74) discovered an increase in activity in the right amygdala, anterior cingulate cortex, and left superior temporal gyrus in psychopathic subjects that were exposed to emotional images with negative valence. However, Zijlmans et al. (84) could not find evidence of amygdalar involvement, but showed that callous-emotional (CU) traits positively correlated with activity in the left superior temporal gyrus and cingulate cortex. Of note, the healthy control group had a greater total psychopathy score than the multi-problem group they were compared to in this study.

The role of amygdala in emotion processing in psychopathy appears inconsistent. Community samples showed that amygdalar hypoactivity was associated with CU traits in processing both positive and negative emotions (100, 118). In contrast, Sadeh and colleagues (119) discovered that impulsive-antisocial dimension of psychopathy positively correlated with amygdalar activity. Moreover, Larson and colleagues (69) found that amygdalar activity did not differ between psychopathic subjects and control group when explicitly attending to a threat. However, psychopathic subjects exhibited decreased fear potentiated startle in terms of reduced amygdalar activity and concomitant increase in activity in the ventrolateral prefrontal cortex and dorsolateral prefrontal cortex when the subjects were engaged in an attentive task prior to presenting the threat (69).

Mier and colleagues (72) investigated the recognition of affective mental states, and found a prominent and widespread hypoactivity in the mirror neuron system of psychopathic subjects, more specifically in the amygdala, inferior prefrontal gyrus, and superior temporal sulcus. Furthermore, psychopathic subjects lacked connectivity between the superior temporal sulcus and amygdala (72). Consistent with a dysfunctional mirror neuron system, Sommer and colleagues (81) discovered that psychopathic subjects exhibited increased activity in attention- and outcome-related areas, including the orbitofrontal cortex, temporoparietal junction, and medial prefrontal cortex, whereas the control group exhibited increased activity in areas associated with empathy and the mirror neuron system including the superior frontal gyrus and supramarginal gyrus. Converging results were found by Reniers and colleagues (120) showing that higher degree of psychopathic traits entailed similar activity changes in areas involved in empathy and moral decision making including the inferior parietal lobule, supramarginal gyrus, precuneus, dorsolateral prefrontal cortex, and medial prefrontal cortex.

In response to pain depicting scenarios, psychopathic subjects showed attenuated activation of also other empathy-related regions including ventromedial prefrontal cortex, periaqueductal gray matter (PAG), posterior superior temporal sulcus (pSTS), and lateral orbitofrontal cortex (54). However, they showed increased activation of mentalizing-related regions including anterior insular cortex, dorsomedial prefrontal cortex, and dorsal striatum (54). Decety, Chen, Harenski, and Kiehl (53) discovered that in psychopathic subjects empathy-eliciting circuits such as the anterior middle cingulate cortex, anterior insular cortex, supplementary motor area, IFG, amygdala, and somatosensory cortex were activated when imaging oneself in pain. However, these circuits were not activated during a third person perspective, i.e. when imagining others in pain. Furthermore, in the third person perspective, psychopathic subjects exhibited an increase in activity in the ventral striatum (53). In fact, activity in the ventral striatum correlated with core psychopathic traits in a similar setting (117). Moreover, Seara-Cardoso, Viding, Lickley, and Sebastian (98) showed that neural responses to imagining others in pain depended on the dimension of psychopathy. More specifically, interpersonal-affective traits negatively correlated with activity in the bilateral anterior insular cortex, IFG, and middle cingulate cortex, whereas antisocial lifestyle traits positively correlated with activity in these areas (98). Molenberghs and colleagues (120) discovered somewhat convergent neural correlates in a punishment setting by showing that a higher degree of psychopathic traits correlated with less activity in brain areas involved in perceiving others in pain, including the anterior insular cortex, orbitofrontal cortex, and dACC. Sitaram and colleagues (122) conducted a pilot study on volitional regulation of the anterior insular cortex by employing negative emotional imageries in conjunction with contingent feedback. They found that one of the four psychopathic subjects learned to regulate the anterior insular cortex.

#### Reward Circuitry

A number of studies reported aberrancies in the reward circuitry in psychopathy. Hosking and colleagues (65) showed that increased subjective value-related activity in the right nucleus accumbens was associated with psychopathy. Furthermore, psychopathic subjects exhibited decreased functional connectivity between ventromedial prefrontal cortex and nucleus accumbens, and this functional connectivity was inversely associated with the frequency of criminal convictions. These findings were ascribed to the interpersonal-affective dimension of psychopathy in particular (65). In contrast, Korponay and colleagues (35) discovered several resting-state functional connectivity aberrancies driven by the lifestyle-antisocial dimension including striato-midbrain, striatostriatal, and corticostriatal connectivities with the latter including increased connectivity between the dorsolateral prefrontal cortex and nucleus accumbens. In a similar vein, Geurts and colleagues (60) found that psychopathic subjects showed increased reward expectancy related activity in the ventral striatum, attributed to impulsive-antisocial traits. Psychopathic subjects also exhibited decreased reward expectancy related activity in the PAG, and increased functional connectivity between the dorsomedial prefrontal cortex and ventral striatum (60). Pujara and colleagues (78) discovered, however, that all dimensions of psychopathy were associated with increased activity in the ventral striatum in a gain versus loss condition. Convergently, reward anticipation in psychopathy correlated with activity in the nucleus accumbens and anterior mesofrontal cortex (50). In a similar setting, Buckholtz and colleagues (123) attributed the increased activity in the nucleus accumbens to antisocial and impulsive traits. As an alternative to monetary rewards, Cope and colleagues (52) approached the setting from a different angle. They presented imprisoned substance-dependent psychopathic subjects drug-related cues and discovered that psychopathy was associated with decreased activity in the anterior cingulate cortex, posterior cingulate cortex, amygdala, hippocampus, globus pallidus, caudate, and frontal gyri.

### Diffusion Tensor MRI Findings in Psychopathy

The integrity of white matter structures appeared to play a pivotal role in psychopathy. Several studies showed reduced fractional anisotropy (FA) in the uncinate fasciculus on the right side (73, 87, 89) and bilaterally (16, 85, 88). These findings were in general attributed to the interpersonal-affective dimensions (85, 89, 124), but also to a lesser extent to lifestyle-antisocial dimensions (85). Moreover, increased radial diffusivity (RD) in the uncinate fasciculus correlated with the interpersonal dimension of psychopathy (16).

In addition to the uncinate fasciculus, aberrancies in other various white matter structures were reported. Sethi and colleagues (86) found that psychopathic subjects exhibited decreased FA in the left dorsal cingulum, indicative of emotional detachment and dysfunction of the default mode network. In turn, Hoppenbrouwers and colleagues (85) discovered bilaterally decreased FA in the uncinate fasciculus, anterior thalamic radiation, and inferior fronto-occipital fasciculus. Furthermore, Yoder, Porges, and Decety (83) conducted a tractography of the amygdalar subnuclei and found that CU traits negatively correlated with functional connectivity between dACC and the central amygdalar subnucleus (83).

### Structural Gray Matter Findings in ASPD

Similar to psychopathy, ASPD was associated with gray matter aberrancies in the limbic and cortical areas. Decreased GMV was noted in anterior cingulate cortex (125–127) superior temporal sulcus, superior temporal gyrus, frontal gyri (125), orbitofrontal cortex (125, 127, 128), sensory motor area, frontopolar cortex (127), medial prefrontal cortex (127, 128), and rectal gyrus (128). In a study by Kolla et al. (110), psychopathic and ASPD groups were compared to each other, and psychopathic individuals had a more pronounced decrease in GMV in temporal and cerebellar regions. Further, contrary to findings in psychopathy, several of the regions with gray matter reductions were accompanied by increased surface area, most notably in the superior temporal gyrus, superior frontal gyrus, superior temporal sulcus, supramarginal gyrus, orbitofrontal cortex, insula, and parahippocampal gyrus (125). Furthermore, ASPD subjects had lower right thalamic volume compared to healthy controls (129). Both the volume of the anterior cingulate cortex and that of the right thalamus negatively correlated with psychosocial deprivation (126, 129). Moreover, increased GMV and WMV were found in ASPD subjects in the inferior parietal lobule and precuneus, respectively (130). However, a study by Howner et al. (34) showed that ASPD individuals had a decreased global brain volume compared to healthy controls.

### Functional MRI Findings in ASPD

Tang et al. (131) investigated resting-state neural activity in ASPD and found that ASPD subjects showed decreased activity in the posterior cerebellum and middle frontal gyrus (MFG). Contrariwise, ASPD subjects showed increased activity in the middle occipital gyrus, inferior temporal gyrus, and inferior occipital gyrus (130). Similarly, Liu, Liao, Jiang, and Wang (132) found decreased activity in the posterior cerebellum, but also in temporal areas and in the orbitofrontal cortex. Recently, Kolla and colleagues (133, 134) noted that monoamine oxidase A (*MAOA*) genotype was associated with ASPD. High activity *MAOA* subjects showed increased resting-state functional connectivity between caudate, frontopolar cortex, and anterior cingulate cortex compared to low activity *MAOA* subjects and healthy controls. The researchers also found that instrumental aggression and functional connectivity from the ventral striatum to the precuneus had an inverse correlation in the low activity *MAOA* subjects, and a positive correlation to the angular gyrus (134). Increased corticostriatal resting-state connectivity was also described in psychopathic individuals (35).

Aberrant neural correlates were also found at task. Firstly, Kumari et al. (135) found that ASPD individuals showed decreased activity in the left frontal gyrus, anterior cingulate cortex, and precuneus in an n-back setting. Secondly, decreased activity in the thalamus was noted in a NoGo condition suggesting impaired control inhibition (136). In a similar vein, Schiffer et al. (97) discovered in a Stroop color naming task that response times and activity in the dorsolateral prefrontal cortex correlated with impulsivity. Furthermore, the ASPD group showed decreased activity most prominently in the left dACC and Wernicke's area compared to healthy controls. Importantly, the decreased activity in the dACC was associated with interpersonal-affective dimensions of psychopathy (97). However, Gregory et al. (62) found more divergent neural activity in ASPD and psychopathy in a reversal learning setting. The researchers found that psychopathic individuals responded to punishment with increased activation of the posterior cingulate cortex and insula, whereas ASPD subjects showed decreased activity in these areas (62). Further evidence for divergent neural correlates was provided by Murray, Shaw, Forbes, and Hyde (96) who showed that antisocial behavior, but not CU traits, was associated with decreased activity in the ventral striatum and dorsolateral prefrontal cortex during reward anticipation.

In a facial emotion processing condition, Hyde, Votruba-Drzal, Hariri, and Manuck (66) found that ASPD traits positively correlated with amygdalar activity, whereas with psychopathic traits the correlation was negative. Indeed, amygdalar hyperreactivity was linked especially to reactive aggression (137). Furthermore, ASPD traits positively and psychopathic traits negatively correlated with tendency to feel unpleasant emotional states (66). In recognizing emotional states based on eyes only, Schiffer and colleagues (138) found no group differences in performance in ASPD versus healthy controls. Contrariwise, psychopathy was associated with diminished ability to recognize emotions. However, ASPD subjects did exhibit decreased activity in the amygdala and increased activity in the left medial prefrontal cortex, ventrolateral prefrontal cortex, pSTS, temporoparietal junction, fusiform gyrus, and precuneus (138).

### Diffusion Tensor MRI Findings in ASPD

Akin to psychopathy, decreased FA was seen in the uncinate fasciculus, inferior fronto-occipital fasciculus, and anterior thalamic radiation in ASPD (88, 139). Of note, axial diffusivity (AD) and radial diffusivity (RD) revealed additional regions not detected with FA alone, implying abnormal axonal structure and demyelination in ASPD, respectively. Importantly, impulsivity negatively correlated with AD in the corpus callosum, posterior corona radiata, and posterior thalamic radiation, whereas risky behavior positively correlated with RD in the superior longitudinal fasciculus and inferior fronto-occipital fasciculus (139). Moreover, the antisocial lifestyle dimensions of psychopathy were associated with decreased FA and increased mean diffusivity (MD) in the frontal lobe (88).

### Structural Gray Matter Findings in CD

CD was associated partly with similar GMV reductions as were seen in psychopathy including amygdala, insula, dorsomedial prefrontal cortex, orbitofrontal cortex, fusiform gyrus, and inferior and superior occipital cortex (31). Dissimilar to psychopathy, a decrease in caudate GMV and an increase in frontal operculum and inferior temporal gyrus GMV was seen. However, the researchers also noted that CU traits positively correlated with the caudate nucleus and ventral striatum consistent with findings of increased striatal volumes in psychopathy. Furthermore, compared to healthy controls, the GMV changes in CD were similar irrespective of childhood- or adolescence on-set with the exception of adolescence on-set group showing GMV reductions in the orbitofrontal cortex. (31). In turn, Budhiraja et al. (140) investigated the brain structure of young women with prior CD diagnosis. They noted an increase in GMV in the superior temporal gyrus and a decrease in GMV in the anterior cingulate cortex, hippocampus, and lingual gyrus, which were attributed mainly to substance use disorder (SUD), anxiety, and depression symptoms (140). In comparison, decreased GMV in both the anterior cingulate cortex and the superior temporal gyrus were reported in psychopathy. Moreover, the findings of Budhiraja et al. (140) may also imply gender specific changes in CD as the sample in Fairchild et al. (31) only included males. Indeed, Lindner et al. (124) emphasized that CD in males and females differed in terms of genotype and phenotype. However, a study by Cohn et al. (90) found reduced gray matter concentration in the insula and amygdala irrespective of gender. CU traits negatively correlated with these findings (90), which are in line with findings in psychopathy. Similarly, a longitudinal cohort study by Pardini, Raine, Erickson, and Loeber (42) showed that decreased bilateral amygdalar volume was associated with higher levels of psychopathy from childhood to adulthood. Furthermore, the amygdalar volumes successfully predicted increased psychopathic features and committing violent acts in a 3-year follow up. The researchers underscored the possibility of amygdalar lateralization by showing that the left amygdalar volume negatively correlated with the lifestyle dimension of psychopathy, while the right amygdalar volume negatively correlated with interpersonal-affective dimensions (42). Moreover, prior CD diagnoses were shown to predict decreased amygdalar activity, higher CU traits and increased aggression as well as impulsivity in adulthood (141).

### Functional MRI Findings in CD

The few functional neuroimaging studies of CD included in this review yielded results quite similar to those in psychopathy. Firstly, resting-state functional connectivity analysis revealed aberrancies in the default mode and salience networks, and also in the frontoparietal network. CU traits were associated with increased connectivity in the left frontopolar cortex within the default mode network. In turn, impulsivity was associated with increased connectivity in the left IFG within the frontoparietal network as well as the left amygdala within the salience network (93). Secondly, Ewbank and colleagues (58) investigated facial emotion processing in CD and found that CD subjects showed decreased amygdalar activity compared to healthy controls. Moreover, psychopathic traits were associated with reduced connectivity between the ventral anterior cingulate cortex and the left amygdala (58).

### Diffusion Tensor MRI Findings in CD

In a study by Lindner et al. (124), young women with a prior CD diagnosis exhibited reduced AD in the forceps minor and the genu and the body of corpus callosum compared to comorbidity matched controls without CD. Furthermore, the researchers could not ascertain abnormal FA in the uncinate fasciculus (124), as was seen in psychopathy on the contrary. However, Pape and colleagues (102) found a positive correlation between FA and with grandiose-manipulative traits in the uncinate fasciculus, corpus callosum, inferior fronto-occipital fasciculus, corticospinal tract, forceps minor, and anterior thalamic radiation in a mixed sample, albeit a non-categorical one. The direction of correlation was negative for RD in the same tracts and a number of other WM tracts. Further, they found that CU traits positively correlated with AD in the corticospinal tract (102). In a similar vein, Passamonti et al. (142) discovered that CD subjects had increased FA, increased AD and decreased RD bilaterally in the external capsule and uncinate fasciculus compared to healthy controls. As this was a male sample, the findings of Lindner et al. (124) may imply gender differences in CD.

## Discussion

The aim of this study was to conduct a systematic literature review on MRI neuroimaging of psychopathic traits, to summarize findings from different MRI modalities that cover different aspects of neural function and structure, and to examine whether these aspects were consistent. A total of 118 records were included in the study. The records consisted mainly of neuroimaging of clinical psychopathy, but also of non-clinical psychopathic traits, antisocial personality disorder, and conduct disorder. Both structurally and functionally, most aberrancies were described in frontotemporal regions as well as in limbic and paralimbic structures.

Psychopathic individuals exhibited decreased GMV in frontotemporal, limbic, paralimbic, and cerebellar structures. Although findings indicated both reduced GMV and abnormal morphology of the hippocampus, evidence for enlargement of the temporal horns in psychopathy was not found nor was it investigated in particular. The temporal horns of the lateral ventricles lie adjacent to the hippocampi. Thus, decreased volumes in hippocampi can inversely correlate with that of temporal horns (143). Temporal horn enlargement has been implicated in some psychiatric diagnoses including Alzheimer disease (144) and schizophrenia (145). Moreover, global GMV of psychopathic individuals does not appear to significantly differ from that of general population (19).

Dysfunction of the default mode network was found. This was anticipated as the default mode network consists of areas overlapping the limbic and paralimbic regions including the temporoparietal junction, posterior cingulate cortex, precuneus, and medial prefrontal cortex (92, 146). Certainly, these regions exhibited decreased GMV, activity, and functional connectivity in psychopathic subjects. The dysfunctional default mode network could, to a degree, relate to the aberrant behavior displayed in psychopathy as the normal function of the default mode network is associated with reflective self-awareness (104), emotional reflection (105), moral judgment (106, 107), and the ability to relive past experiences and construct possible futures (147). However, ASPD was also associated with dysfunction in several networks including default mode (134), attention, cerebellar (131), and frontoparietal control networks (148).

Furthermore, findings from DTI studies corroborate the aforesaid notions. The uncinate fasciculus was the white matter tract with most anomalies in terms of decreased FA. The uncinate fasciculus connects the amygdala to ventromedial prefrontal cortex and orbitofrontal cortex and is ostensibly responsible for several cognitive and affective functions that are erring in psychopathy including moral judgment, empathy, value representation, and stimulus-reinforced learning (14, 149, 150). However, reduced FA in the uncinate fasciculus cannot be considered strictly specific to psychopathy as similar findings were reported in ASPD and have previously been reported in patients with generalized anxiety disorder (151) and major depression disorder (152). Notwithstanding, reduced FA seems to be a viable marker for affective and social disorders. Another white matter tract implicated in psychopathy was the dorsal cingulum that connects posterior cingulate cortex to medial prefrontal cortex and is associated with social and emotional cognition (153). Decreased FA in this tract was associated with interpersonal-affective dimensions of psychopathy and emotional detachment. As similar findings have been reported in other psychiatric conditions such as post-traumatic stress disorder (154) and schizophrenia (155), reduced FA in the dorsal cingulum is also not specific for psychopathy. Moreover, Hoppenbrouwers and colleagues (85) suggest that a dysfunctional striato-thalamo-frontal network and mesolimbic reward system is present in psychopathy. Yoder, Porges, and Decety (101) postulate further in their tractography study that different psychopathic traits may arise from different parts of the highly specialized amygdala.

These findings are in accordance with the recently proposed Impaired Integration Theory (IIT) (156). The IIT attempts to integrate psychopathic manifestations, such as emotional detachment and impaired ability to incorporate perceived information into operant and contextual learning, with brain abnormalities inherent to psychopathy (156).

Interestingly, empathy-related regions in the brain were active in psychopathic subjects when imagining oneself in pain (53). However, when imagining others in pain, these areas were not active. This being said, psychopathic individuals appear not to lack the apparatus for empathy, yet they are evidently unable to simulate and understand the internal states of others. Moreover, the activation of ventral striatum in imagining others in pain might indicate that psychopathic individuals take pleasure in observing others in pain (53). Furthermore, the recognition of the affective mental states of others in psychopathy was attributed to decreased activity in the mirror neuron system (MNS) and increased activity in outcome-related regions (72, 81). A similar compensation mechanism for deficient empathy by engaging more cognitive areas of the brain was also seen in ASPD (138). Concisely, the MNS represents a mechanism by which the motor processes and representations of one individual displaying a motor function can be induced in another individual by merely observing the first individual (157). However, such a mimicry is likely insufficient to understand the emotions or actions of others (157), which is a complex cognitive process involving the Theory of Mind (ToM) comprising areas significantly overlapping with the default mode network (158). Dysfunction of the MNS has also been reported in autism spectrum disorders (159).

Psychopathic individuals display lack of empathy and affective cognition, and they might even be unconquerable by love. The mesolimbic reward system, together with limbic and paralimbic system, contribute to the feeling of romantic love (160). All these three systems were dysfunctional in psychopathy. In addition, according to a recent qualitative study of former spouses to psychopathic individuals per the PCL-R, the former spouses were repeatedly subjected to coercion, conning, and manipulation (161). We speculate that psychopathic individuals might not be capable of romantic love, based on the notion that love and desire are two neuroanatomically and fundamentally separate entities (160). Data on this topic are scarce, and the topic opens up interesting opportunities for future studies.

Also, intriguingly, aberrant cerebellar function and structure were reported in psychopathy. Beyond the cerebellum's traditional role in motor functions, an increasing amount of evidence indicates that the cerebellum has functions pertaining to emotional and cognitive control as well as morality (162–164). Schmahmann (165) posits that the cognitive and limbic functions of the cerebellum lie in the posterior lobe, in line with the findings in this review. Firstly, the posterior cerebellar lobe exhibited reduced activity in a moral judgment task in psychopathic subjects (114). Secondly, emotion recognition was associated with increased GMV in the posterior cerebellar lobe (76). Thirdly, reduced resting-state activity in the posterior cerebellum was found in ASPD subjects (131, 132). Moreover, lesion studies have shown that damage to the posterior cerebellar lobe can lead to deficient cognitive and affective information processing (166). We suggest that a deeper investigation into the role cerebellum in psychopathy is warranted and might result in new insights.

Only one of the imaging studies focused on a specific genotype and its relationship to ASPD (134). Twin studies suggest that heredity play a pivotal role in psychopathic traits across childhood (167–170), adolescence (171–175), and adulthood (172, 177, 178). Up to 70% of the variance in psychopathic traits may be attributable to genetics according to recent studies (177–180). However, the involved genes remain to be identified (180). One noteworthy candidate is the human serotonin transporter gene (*SLC6A4*) (181, 182). *SLC6A4* manifests in two forms, and carriers of the short allele are predisposed to negative mental health aspects including anxiety, depression, substance use disorder, and suicide (181), whereas homozygosity of the long allele is associated with emotional detachment and psychopathic traits (182). Another candidate is the X-linked monoamine oxidase A (*MAOA*) gene and its high (*MAOA-H*) and low activity alleles (*MAOA-L*) (183). Individuals with absent or low acting *MAOA* are more prone to aggressive and impulsive behavior and exhibit higher psychopathic traits (184). Furthermore, identifying genes may reveal viable biomarkers for psychopathy. Recently psychopathy was also associated with upregulation of Ribosomal protein L10 Pseudogene 9 (*RPL10P9*), Zinc finger protein 132 (*ZNF132*), and downregulation of Cadherin-5 (*CDH5*) and Opioid receptor Delta 1 (*OPRD1*) genes, which explained 30% to 92% of the variance in psychopathic symptoms in a stem cell derived study by Tiihonen et al. (185). Identifying more genes and examining their relationship to brain structure and function might provide useful information of the neurobiological etiology of psychopathy. Some of the variance seen in genetic or proteomic studies might also be visualizable with modern or upcoming imaging techniques.

Discovering viable biomarkers for psychopathy is challenging. The results in this review suggest that psychopathy and ASPD might stem from dissimilar biological processes and show divergent neural correlates, yet antisociality and core features of psychopathy are clumped into one disorder. The hypothesis of divergent neural correlates explains not only why some heterogeneity was seen in neuroimaging results of psychopathy, but also why there were many similar anomalies in ASPD and psychopathy (**Table 3**). For example, Sato and colleagues (45) managed to discriminate psychopathic subjects from healthy controls based on gray matter changes, but the psychopathic subjects all had comorbid ASPD. Further, even though Sadeh et al. (119) did not find a correlation between core psychopathy and amygdalar hypoactivity, the researchers emphasize, however, that this finding is not in direct contradiction with the theory of amygdalar hypoactivity in psychopathy, but rather that it provides evidence of divergent neural correlates with respect to more general antisociality and core psychopathy. It has, however, also been suggested that ASPD is a subtype of psychopathy (186). Furthermore, these two conceptually dissimilar notions are occasionally used arbitrarily (187). Emphasizing both the inconsistent use of the terms and the dissimilarities between antisociality and psychopathy, ASPD has aptly been described as “a euphemism par excellence” [(188) p. 301]. Taking the aforesaid into consideration, it is difficult to reach high specificity for potential biomarkers for core psychopathy unless interpersonal-affective and lifestyle-antisocial dimensions are considered separately. To play with the thought and try to ensnare core psychopathy, the following combination of tests could be attempted. Firstly, a DTI showing reduced FA in the uncinate fasciculus, possibly accompanied by increased RD. Secondly, an fMRI with an affective mental state recognition or empathy-related task showing both an increase in activity in outcome and attention-related areas and a concomitant decrease in activity in the MNS and ToM.

**Table 3:** Key dissimilarities and similarities between core psychopathy and ASPD.

Setting/Characteristic	Core psychopathy	ASPD
CU traits/Emotional dectachment	Always	Often comorbid
Empathy compensation with executive brain regions	Yes	Yes
Facial emotion recognition	Amygdalar hyporeactivity	Amygdala hyperreactivity
Emotion recognition ability	Diminished	Normal
Response to punishment	Increased activity in limbic/paralimbic areas	Decreased activity in limbic/paralimbic areas
Decreased GMV in limbic/paralimbic areas	Yes	Yes
MAOA -related	No	Yes
Hereditary	Highly	Moderately
Aggression	Proactive/instrumental	Reactive
Notably negative emotionality	No	Yes
WMT aberrancies	Decreased FA in the UF	Decreased FA in the UF
DMN dysfunction	Yes	Yes

CU, callous-unemotional; FA, fractional anisotropy; GMV, gray matter volume; MAOA, monoamine oxidase A; UF, uncinate fasciculus; WMT, white matter tract.

There has been increasing interest to understand and discover the neural correlates of psychopathy during the past years. Although certain noteworthy patterns and neural correlates have frequently transpired, the neurobiological etiology of psychopathy remains obscure. Furthermore, the findings suggest that “successful” psychopathic individuals may not show similar structural gray matter changes as their “unsuccessful” counterparts. Consequently, if the single thing separating “successful” psychopathy from “unsuccessful” psychopathy is a criminal conviction, then a vast amount of neuroimaging data is yet to be obtained. The majority of the neuroimaging studies are conducted in forensic or prison-related settings, and these unsuccessful psychopathic individuals “represent only the tip of a very large iceberg” [(189) p. 115]. Therefore, focusing on non-clinical and community settings could facilitate the unraveling of the etiology of psychopathy.

This review has several strengths. Firstly, three MRI submodalities were included in this study. Secondly, neuroimaging studies of psychopathic traits in community and clinical settings were included in addition to forensic and prison populations. Thirdly, we included studies with both genders in this review. Fourthly, we strived to include a number of adolescence studies as well, as psychopathic traits manifest as a continuum from childhood to adulthood.

The qualitative synthesis was not without challenges. Firstly, a plethora of different tasks were seen in functional neuroimaging. These tasks needed to be grouped to be able to provide a coherent written summary. Furthermore, some compromise between the readability and high level of details needed to be made, although the Review Matrix contains findings in a more detailed level. Secondly, psychopathy and psychopathic traits have both various definitions and instruments to measure them. Including other instruments apart from the PCL-R can be seen both as a limitation and strength. On the one hand, this can hinder the generalizability of the results. On the other hand, more studies in various settings met the inclusion criteria due to this decision. Further, several of the non-PCL-R instruments are cross-validated with the PCL-R.

Perhaps the most challenging aspect of this review was taking into consideration the high comorbidity of the trait continuums of ASPD, CD, and psychopathy. These heritable disorders reflect independent structural and functional aberrancies in the brain, but also seem to manifest convergent biological processes to some extent. For example, both CD and ASPD are related to dysfunction of the default mode network (93, 130) and to decreased GMV in limbic and cortical regions (31, 128). Further, the said disorders are not mutually exclusive nor are they biologically dichotomic constructs. These confounders and the arbitrary use the notions of psychopathy and ASPD call for coherence and attentiveness in future research. Another comorbidity of note is substance abuse disorder, which damages the integrity of white matter (190, 191), and induces volumetric gray matter reductions (192, 193).

Another limitation of note is that this review focused on MRI submodalities. A review on PET, SPECT, and EEG could shed light on the abstruse neurobiological etiology of psychopathy, and even add support to our findings. The age criterion applied in this review comprises a limitation as it led to the exclusion of several studies. As such, psychopathic traits in childhood and adolescence may require a systematic literature review of their own. Moreover, notwithstanding the inclusion of females in this review, the majority of the studies were conducted on males. This warrants caution in generalizing the results and more research on female psychopathy. Further, it is paramount to mention that “the lack of longitudinal neuroimaging means that *persistence* of neural abnormalities can only be inferred, not investigated” as aptly put by Linder [(194) p. 68].

## Conclusions

This systematic review sums that structural and functional aberrancies involving the limbic and paralimbic systems including reduced integrity of the uncinate fasciculus appear to be associated with core psychopathic features. A deeper investigation into the role of the cerebellum in psychopathy is also warranted and might result in new insights. Furthermore, the evidence suggests that ASPD and psychopathy stem from divergent biological processes. Still, more neuroimaging studies are warranted particularly with respect to female and community psychopathy.

## Data Availability Statement

All datasets generated for this study are included in the article/**Supplementary Material**.

## Author Contributions

MJ, ML, and JT conceived the presented idea. MJ wrote the draft of the manuscript and constructed the tables and figures. ML and JT provided critical feedback and helped shape the manuscript. ML and OV assisted MJ in reviewing the records as specified in the Materials and Methods section. MJ and ML calculated the Cohen's kappa.

## Funding

This study was funded by the Finnish Ministry of Social Affairs and Health through a developmental fund for Niuvanniemi Hospital.

## Conflict of Interest

ML is a major share holder and board member at Genomi Solutions Ltd., a Finnish bioinformatics company. He has also received grants or honoraria from Sunovion Ltd. and Orion Pharma Ltd. and research scholarships from the Finnish Cultural Foundation and Finnish Medical Foundation. JT has received personal fees from the Finnish Medicines Agency (Fimea), AstraZeneca, Bristol-Meyers Squibb, Eli Lilly, F Hoffman-La Roche, GlaxoSmithKline, Janssen-Cilag, Lundbeck, Novartis, Organon, Otsuka, and Pfizer; and has received grants from the Stanley Foundation, Sigrid Jusélius Foundation, Eli Lilly, and Janssen-Cilag.

The remaining authors declare that the research was conducted in the absence of any commercial or financial relationships that could be construed as a potential conflict of interest.
