# Supplementary material for: A Systematic Literature Review of Neuroimaging of Psychopathic Traits
Source: Front Psychiatry. 2020 Feb 6;10:1027. doi: 10.3389/fpsyt.2019.01027 (PMC7016047; doi:10.3389/fpsyt.2019.01027)
Supplement: Supplementary file 1 [file DataSheet_1.docx]

Supplementary Material

This document contains Supplementary Tables S1-S5 and the search strings used in the data collection processes.

Supplementary Table 1: Study characteristics and key findings for neuroimaging studies of psychopathic traits

| **No.** | **Record** | **Type & Design** | **Sample Characteristics** | **Exclusion Criteria & Covariates** | **Behavioral Measures** | **Method** | **Key Findings** |
| --- | --- | --- | --- | --- | --- | --- | --- |
| **1** | Boccardi et al. (2010) | 4 P (34.6) v. MP (25.9) v. HC | 12 P (0) 33.0 14 MP (0) 32.1 25 HC (0) 34.6 | Previous mental disorder or behavioral disorder caused by brain damage.  Covariates: Cloninger type 2 alcoholism, polysubstance abuse; subjects divided into groups of high and medium psychopathy | AB: ASPD inmates on pretrial forensic evaluation and charged with violent offences; DSM-IV (I); ICD-10 (I)  P: PCL-R (I) | 1.0T MRI ROI-M VBA | P showed in the hippocampi anteriorly a GM reduction of 20%, a depression on the longitudinal axis, and enlargement of the lateral borders. Negative correlations with PCL-R score and the posterior hippocampal sectors. |
| **2** | Tang et al. (2013) | 2 ASPD v. HC | 32 ASPD (0) 20.5 34 HC (0) 21.67 | Previous mental disorder or behavioral disorder caused by brain damage.  Covariates: Age, IQ and education matched HC; reward | AB: youth offenders with ASPD; DSM-IV (I); PDQ (S) | 1.5T fMRI; rest Whole brain and ROI VBA | ASPD showed decreased regional homogeneity in the right CB posterior lobe and in the right MFG; and increased regional homogeneity in the right MOG, left ITG, and the right IOG. |
| **3** | Osumi et al. (2012) | 1 NC (33.6) | 20 S (0) 19.5 | N/A  Covariates: N/A | AB: successful undergraduate psychopaths  P: LP (S) | 1.5T fMRI; task Whole brain VBA | Psychopathy was negatively correlated with activity in the right amygdala, thalamus, and the left SPL in unfair vs. fair contrast.  Psychopathy was negatively correlated with activity in the right putamen/lateral GP, the right MTG, and the left STG in response to punishment.  Psychopathy was negatively correlated with functional connectivity between amygdala and putamen, substantia nigra, subthalamic nucleus, red nucleus. NAcc, STG, AIC. |
| **4** | Hyde, Byrd, Votruba-Drzal, Hariri, & Manuck (Hyde et al., 2014) | 1 P (285) v. ASPD | 103 S (55) 44.6 | Conditions potentially affecting blood flow; history of psychotic symptoms; psychotropic medications; DSM-IV Axis 1 disorders  Covariates: N/A | AB: NEO-PI-R (S); MPQ-BF (S)  P: PCL-R (C) , PPI (C) | 3T fMRI; task ROI-M VBA | ASPD traits positively and psychopathic traits negatively correlated with amygdala reactivity. ASPD traits positively and psychopathic traits negatively correlated with low negative emotionality i.e. tendency to feel unpleasant emotional states. |
| **5** | Yoder, Porger, & Decety (2015) | 1 NC (307.82) | 43 S (0) 25 | N/A  Covariates: mixed martial arts interest | P: PPI-R (S) | 3T fMRI and DTI; task Whole brain PT | Viewing mixed martial arts: Fearless Dominance scores inversely correlated with activity in the right STC, overlapping with the right TPJ. Fearless Dominance correlated positively with functional connectivity between right BLA and regions in bilateral occipital, fusiform, temporal, and parietal cortex; between CE and left posterior INS and CB. Not significantly related to connectivity seeded with the full right amygdala.   Coldheartedness was negatively related to activity in bilateral occipital cortex and CB, as well as left MFG. Coldheartedness negatively correlated with connectivity between CE and the left dorsal ACC.  Self- Centered Impulsivity positively correlated with activity in bilateral caudate and predicted decreased coupling between BLA and left IFG. |
| **6** | Decety, Chen, Harenski, & Kiehl (2013) | 4 P (≥30) v. MP (21-29) v. C (≤ 20) | 37 P (0) 32.5 44 MP (0) 34.1 40 C (0) 34.6 | Age < 18 or > 55, non-fluency in English, reading level lower than 4th grade, IQ < 80, history of seizures, prior head trauma with loss of consciousness > 30 min, DSM-IV Axis I diagnosis, lifetime history of psychotic disorder, psychotic disorder in a first degree relative, current alcohol or drug use.  Covariates: Subjects divided to groups of high (P), medium (MP), and low psychopathy (C); age, IQ, ethnicity, and DSM-IV Axis II comorbidity matched groups. | AB: male offenders from a medium-security correctional facility  P: PCL-R (I) | 1.5T fMRI; task ROI-A VBA | P vs. C showed increased activity in the anterior MCC, SMA, right AIC, IFG and right posterior STS/TPJ during imagine self in pain perspective. PCL-R total scores and F2 positively correlated with activity in right anterior insula. F1 and F2 negatively correlated with dmPFC activation.  P showed increased connectivity between AIC and the right posterior STS as well as positive coupling with the posterior STS/TPJ, ventral PFC, mPFC, and dlPFC. C showed decreased connectivity between AIC, hippocampus, and OFC as well as negative coupling between the amygdala and ventral PFC and mPFC.  P vs. C showed increased activity in the dlPFC and VS (right caudate) during imaging other in pain perspective. PCL-R total scores, F1, and F2 negatively correlated with activity in right AIC. F1 positively correlated with VS activity and negatively correlated with activity in right amygdala. F1 and F2 positively correlated with dmPFC activation.   P showed negative connectivity between the anterior insula and right OFC and PCC as well as negative coupling with the OFC and dlPFC. C showed positive connectivity between the anterior insula and PCC and dlPFC as well as positive coupling with the OFC. |
| **7** | Seara-Cardoso et al. (2016) | 1 NC1 (54.68) NC2 (52.20) | 28 S1 (0) 26.3 28 S2 (0) 23.0 | Neurological or psychiatric disorders  Covariates: The study consisted of two substudies with their respective samples; age | P: SRP-SF (S) | 1.5T fMRI; task ROI-A VBA | Processing potential personal everyday moral transgressions elicited responses in right SMG, bilateral amygdala, bilateral AIC, and vmPFC across all groups.   Left AIC modulation of anticipated guilt was weaker in individuals with higher levels of interpersonal psychopathic traits. |
| **8** | Shao & Lee (2017) | 1 P (336.97) v. C (267.84) | 29 P (49) 20.14 23 C (52) 20.04 | Age < 18, a history of major physical illness, neurological or psychological conditions, such as psychotic disorders, affective disorders or addictions in self or first degree relative, current medication, non-corrected visual impairment.  Covariates: subjects divided to high and low psychopaths | P: PPI-R (S) | 3T fMRI ROI-A VBA | P showed lying-related activity reductions in dlPFC, vmPFC, ACC, and CB compared to C. |
| **9** | Glenn, Han, Yang, Raine, & Schug (2017) | 1 NC (22.7) | 16 S (12.5) 30.2 | Age < 18, nonfluency in English, a history of epilepsy.  Covariates: ethnicity | P: PCL-R (I) | 3T fMRI; task ROI-A VBA | Lie vs. truth main contrast: activation in the right dlPFC, right FPC, and the right IPL, including the SMG and AG.   Total PCL-R score positively correlated with activity in the FPC, SMG, insula, and lateral frontal cortex. F1 negatively correlated with activity in the left dlPFC.   Autobiographical lie vs. truth: Total PCL-R score positively correlated with activity in the lateral frontal lobe bilaterally, insula, FPC, and SMG. Total PCL-R score negatively correlated with activity in the OFC, AG, and dlPFC. F1 correlated positively with activity in the bilateral lateral frontal lobe and insula. F2 correlated positively with activity in the FPC and SMG. F1 and F2 correlated negatively with activity in the OFC.   Non-autobiographical lie vs. truth: total PCL-R score positively correlated with activity in the FPC and SMG, and negatively with dACC and insula.   Criminal informational lie vs. truth: total PCL-R score positively correlated with activity in the ACC, dlPFC, insula, and FPC. |
| **10** | Lindner et al. (2018) | 1 NC (5.22) | 73 S (100) 24.6 | N/A  Covariates: drug dependence, depression, anxiety disorder, psychoactive medication, IQ; 43 of subjects with history of substance misuse in adolescence | P: PCL-R (I) | 3T fMRI; rest ROI-A | Positive correlations in connectivity of intra- and interhemispheric regions and total PCL-R scores were detected between right caudate and right MFG; right caudate and left rectus; left SMA and right rectus; left STP and right paracentral lobule. Factor 2 positively correlated with left SMA and left rectus; left SMA and right rectus.   Total PCL-R scores were negatively associated with the strength of the left inferior TPJ and positively associated with betweenness of the right TPJ. Total psychopathy scores also negatively correlated with efficiency of the right AG. F1 positively correlated with right insula and right hippocampus betweenness.   Impaired Integration Theory predictions of abnormal topology in Default Mode Network nodes were confirmed in a sample of females with PCL-R measured psychopathic traits. |
| **11** | Boccardi et al. (2013) | 4 P (29.9) v. HC | 26 P (0) 32.5 25 HC (0) 34.6 | Previous mental disorder or behavioral disorder caused by brain damage  Covariates: Cloninger type 2 alcoholism, polysubstance abuse | AB: persons on pretrial forensic evaluation and charged with violent offences  P: PCL-R (I) | 1.0T MRI ROI-M VBA | P vs. HC showed a 13% GMV reduction bilaterally in NAcc accompanied by abnormal morphology. Normal size, but abnormal morphology in caudate and putamen.  Facet 3 of the PCL-R scale significantly correlated with caudate and putamen morphology. |
| **12** | Leutgeb et al. (2015) | 4 P (20.6) v. HC (1.6) | 40 P (0) 38.1 37 HC (0) 36.7 | S: Depression, bipolar disorder, psychosis, attention-deficit hyperactivity disorder, as well as organic or neurological conditions; substance abuse during imprisonment  HC: conviction for any crime, a history of a mental disorder or substance abuse.  Covariates: Age and educational level matched HC | AB: violent male offenders from a maximum security correctional facility; VRAG (I); STAXI (S);VRS (I)  P: PCL-R (I) | 3T MRI ROI-A VBA | P showed increased GMV in the right CB, right vermis, left caudate, and left GP as well as decreased GMV in the right dmPFC.   F1 correlated with GMV positively in the right CB and negatively in the right dlPFC. F2 correlated with GMV positively in right SMA, right putamen, left pallidum, bilat. OFC and bilat. insula. |
| **13** | Tiihonen et al. (2008) | 4 P (34.6) v. LP (25.9) v. HC | P 12 (0) 33.0 LP 14 (0) 32.1 HC 25 (0) 34.6 | Previous mental disorder or behavioral disorder caused by brain damage  Covariates: Cloninger type 2 alcoholism, polysubstance abuse; ASPD group divided to high (P) and low psychopaths (LP) | AB: ASPD inmates on pretrial forensic evaluation and charged with violent offences; DSM-IV (I); ICD-10 (I)  P: PCL-R (I) | 1.0T MRI ROI-M VBA | P + LP compared to HC showed decreased GMV bilaterally in the postcentral gyri, FPC and OFC, left PCC, and right insula. They also showed increased GMV in the right CB as well as increased WMV bilaterally in the parietal lobes, occipital lobes, and left CB.   In P compared to HC, the same GM areas remained significant. Additionally, P showed GMV reductions in left MTG, and left parahippocampal gyrus.   In LP compared to HC no significant reductions in GMV were observed. |
| **14** | Decety, Skelly, & Kiehl (2013) | 4 P (≥30) v. MP (21-29) v. C (≤ 20) | 27 P (0)  28 MP (0) 25 C (0) | N/A  Covariates: subjects divided to groups of high (P), medium (MP), and low psychopathy (C);  Age, IQ, ethnicity, and DSM-IV Axis II comorbidity matched. | AB: male offenders from a medium-security correctional facility P: PCL-R (I) | 1.5T fMRI; Task ROI-A VBA | Viewing individuals being hurt: P showed increased activation of the SMA, dACC, bilateral AIC, GP, IFG, mPFC, posterior STS, postcentral gyrus and SMG. C showed increased activation of PAG, vmPFC and lateral OFC.  Both F1 and F2 correlated negatively with activity in PAG, vmPFC and superior temporal pole; and positively with right AIC, right IFG, right posterior STS, right SFG, right dmPFC and left precuneus. F1 correlated positively with activity the right SMA, bilateral dACC, bilateral dorsal striatum, IFG and somatosensory cortex.  Viewing facial expressions of pain: P showed increased activity in the AIC, postcentral gyrus, IPL and precentral gyrus. C showed increased activity in the bilateral IFG, MCC, angular gyrus, putamen, pSTS, SMG, dmPFC, GP, dACC, vmPFC, and medial OFC. Both F1 and F2 correlated negatively with activity in MCC, IFG, dmPFC and left AG; and positively with AIC activity. F1 correlated negatively with activity in the right AG, and left pSTS; and positively with left postcentral gyrus and right precentral gyrus. F2 correlated negatively with right STS, dACC, GP, and putamen.   Seeing others in pain regardless of stimulus type: P showed increased activity in the AIC. C showed increased activation of in the right vmPFC and right lateral OFC. |
| **15** | Pera-Guardiola et al. (2016) | 4 P (27.8) v. HC | 19 P (0) 39.2 20 HC (0) 40.6 | Symptomatic medical or neurological disorder; DSM-IV Axis I disorders (except for SUD), Axis II disorders (except for ASPD)  Covariates: SUD; age, gender and vocabular IQ matched HC | AB: violent male offenders from correctional institutes  P: PCL-R (I) | 1.5T fMRI; Task Whole-brain VBA | P showed deficient recognition of sad, happy and fear emotional expressions compared to HC. In P, the recognition of emotional expressions positively correlated with GMV in the OFC, IFG, dmPFC, and somatosensory cortex. In HC, the recognition of emotional expressions positively correlated with GMV in amygdala and temporal cortex. P vs. HC showed decreased GMV only in the posterior insula. No correlations with PCL-R found. |
| **16** | Fairchild et al. (2011) | 1 ECD (2.46) v. ACD (2.47) v. HC (1.98) | ECD 36 (0) 17.69 27 ACD (0) 17.89 27 HC (0) 18.53 | IQ < 80, presence of a pervasive development disorder  Covariates: IQ matched control group; ADHD-symptoms  Subjects divided into adolescent-onset CD (ACD) and early-onset CD (ECD) | AB: persons from Pupil Referral Units and Youth Offending Services; K-SADS (I)  P: YPI (S); ICU (P) | 3T MRI ROI-A VBA | ECD + ACD vs. HC showed reduced GMV in bilateral amygdala, left insula, left dmPFC, bilateral caudate, left fusiform gyrus, left inferior and superior occipital cortex; and increased GMV in left frontal operculum and left ITG.   ACD vs. HC showed reduced GMV in bilateral amygdala, right ventral insula, left OFC and dmPFC.  ECD vs. HC showed reduced GMV in right amygdala.  ACD vs. ECD no findings.   No correlations with total psychopathy score. CU traits positively correlated with GMV in the caudate nucleus and ventral striatum. |
| **17** | Bertsch et al. (2013) | 4 P (23.8) v. HC | 12 P (0) 27.3 14 HC (0) 26.1 | N/A  Covariates: age, gender and IQ matched HC | AB: male ASPD inmates convicted of violent crimes; DSM-IV (I)  P: PCL-R (I) | 1.5T MRI ROI-A VBA | P vs. HC exhibited significantly reduced GMV in the left postcentral gyrus, left dmPFC, right posterior cingulate/precuneus, and bilateral occipital cortex. |
| **18** | Pujol et al. (2012) | 4 P (27.8) v. HC | 22 P (0) 39.8 22 HC (0) 40.6 | DSM-IV Axis I disorders (except for previous substance misuse), DSM-IV Axis II disorders (except for ASPD), non-normal IQ.  Covariates: age, IQ, and gender matched HC. | AB: male inmates convicted of violent crimes P: PCL-R (I) | 1.5T fMRI; Task + rest Whole-brain VBA | Moral dilemma condition: HC showed activation of mPFC, PCC/precuneus, and bilateral AG. P showed overlapping activity, but decreased activity in mPFC and PCC. P vs. HC showed decreased activity in hippocampi, and the posterior-inferior midbrain extending to PAG, locus coeruleus and CB.   P showed decreased functional connectivity at rest in anterior and posterior DMN. The medial frontal region showed functional connectivity reduction with the PCC and nearby visual areas. The PCC also showed reduced functional connectivity with frontal areas, including the mPFC extending to dlPFC and vlPFC.   PCL-R total scores negatively correlated with activity in the PCC and right AG. |
| **19** | Liu, Liao, Jiang, & Wang (2014) | 2 ASPD v. HC | 32 ASPD (0) 20.5 35 HC (0) 21.67 | Previous mental disorder or behavioral disorder caused by brain damage.  Covariates: age, IQ and education matched HC; reward | AB: youth offenders with ASPD; DSM-IV (I); PDQ (S) | 1.5T fMRI; Rest Whole-brain + ROI VBA | ASPD vs. HC showed decreased activity in the right ITG, left temporal lobe, right OFC, left CB posterior lobe. |
| **20** | Lindner et al. (2016) | 1, 3 CD v. CC v. HC | 28 CD (1000) 24.1 15 CC (100) 25.2  HC 24 (100) 22.9 | All: history of bipolar disorder, psychosis, physical handicap or neurological disorder  HC: Axis I and II disorders  Covariates: comorbidity matched control group without CD (CC); Age matched HC; physical and sexual abuse; | AB: females with previous CD diagnosis and history of substance misuse in adolescence; K-SADS (I); DSM-IV (I); MCVI (I) | 3T DTI Whole-brain TBSS | CD v. HC: Reduced axial diffusivity in bilateral CC, Fmin, CG, left IFOF, and left CR.  CD v. CC: Reduced axial diffusivity primarily in the left Fmin and the genu and the body of the CC. |
| **21** | Raine et al. (2003) | 1 P (30.3) v. HC (10.8) | 15 P (0) 31.6 25 HC (0) 28.8 | Age younger than 21 years or older than 45 years, nonfluency in English, history of epilepsy  Covariates: SUD | AB: ASPD males; NYS-AD (S); criminal history transcripts; DSM-IV (I);  P: PCL-R (I); IM-P (I); | 1.5T MRI ROI-M VBA | P vs. HC showed a 22.6% increase in the WM volume of the CC and CR. PCL-R total score positively correlated with CC volume.   P showed increased functional connectivity between the two hemispheres with a positive correlation with Total PCL-R score. |
| **22** | Boccardi et al. (2011) | 4 P (29.9) v. HC | 26 P (0) 32.5 25 HC (0) 34.6 | Previous mental disorder or behavioral disorder caused by brain damage  Covariates: Cloninger type 2 alcoholism, polysubstance abuse | AB: ASPD inmates on pretrial forensic evaluation and charged with violent offences; DSM-IV (I); ICD-10 (I)  P: PCL-R (I) | 1.0T MRI ROI-M VBA | P vs. HC showed GM reductions bilaterally in the OFC, IFG, MFG, SFG, parahippocampal gyrus, ventromedial ACC, and cuneus. Additionally, right frontal pole, left precuneus, right retrosplenial cortex, left motor-sensory cortex, right frontoparietal cortex, and left fusiform gyrus.   P vs. HC showed greater global volumes of the amygdalae accompanied by differences in morphology. |
| **23** | Budhiraja et al. (2017) | 1 CD (0.33) v. HC (0.19) | 31 CD (100) 24.1 25 HC (100) 22.7 | All: history of psychosis, bipolar disorder, autism, or Cluster A or C personality disorder, neurological disorder, loss of consciousness for more than 30 minutes.   HC: CD, Axis I and II disorders, criminal behavior.  Covariates: age matched HC | AB: prior CD diagnosis; K-SADS (I); DSM-IV (I)  P: 25 subjects PCL-YV (I); 6 subjects and HC PCL-SV (I); note: Facet 2 only | 3T MRI Whole-brain and ROI-A VBA | CD vs. HC showed increased GMV in the left STG and reduced GMV in lingual gyrus, left hippocampus, and left ACC. |
| **24** | Ly et al. (2012) | 4 P (31.8) v. C (13.5) | 21 P (0) 32.7 31 C (0) 32.1 | Age > 45, IQ < 70, a history of psychosis or bipolar disorder, current psychotropic medications  Covariates: subjects divided to psychopaths (P) and nonpsychopaths (C) | AB: male offenders from a medium-security correctional facility  P: PCL-R (I) | 1.5T MRI ROI-A VBA | P vs. C showed reduced GM bilaterally in temporal poles and precentral gyri, left medial SFG, AIC, STG, fusiform gyrus, lateral occipital cortex, posterior insula, dACC, lateral SFG, MFG, as well as right pSTG and IFG. |
| **25** | Kolla, Dunlop, Meyer, & Downar (2018) | 1 ASPD (26.4) v. HC (3.1) | 21 ASPD (0) 36.2 19 HC (0) 34.2 | A history of major depressive disorder, bipolar disorder, or a schizophrenia spectrum illness, current nonalcohol drug misuse, use of psychotropic medication, cigarette smoking.  Covariates: IQ; MAO-A genotype and age matched HC | AB: males with criminal record; DSM-IV (I); BPAQ (S); BIS-11 (S); RPQ (S);  P: PCL-R (I) | 3T fMRI; rest ROI-M VBA | ASPD MAOA-H vs. ASPD MAOA-L + HC exhibited increased dorsal caudate functional connectivity to the right frontal pole and bilateral ACC.  Total PCL-R score correlated positively with MAOA-L polymorphism. |
| **26** | Sheng, Gheytanchi, & Aziz-Zadeh (2010) | 1 NC | 19 S (68) 27.7 | A history of neurological or psychiatric conditions  Covariates: N/A | P: PPI-R (S) | 3T fMRI; task and rest ROI VBA | Speech production and event-related paradigm task: PMC and mPFC, parts of the DMN, were less active during task than during a rest. Machiavellian Egocentricity negatively correlated with activity in the posteromedial cortex. Carefree Nonplanfulness negatively correlated with activity in the medial PFC. |
| **27** | Cohn et al. (2015) | 2 NC (83.1) | 130 S (14.6) 17.8 | N/A  Covariates: Subjects recruited from a longitudinal study; ADHD, DBD, PTSD; | AB: persons arrested before the age of 12; NIMH DISC-IV (I); RPQ (S)  P: YPI (S) | 3T fMRI; rest ROI | Callous‐unemotional traits positively correlated with increased within‐network connectivity in the DMN in the left FPC.  Impulsive‐irresponsible traits positively correlated with connectivity in the left IFG within the right frontoparietal network, as well as between the left frontoparietal network and inferior frontal network. Impulsive‐irresponsible traits negatively correlated with connectivity in the left amygdala within the salience network. |
| **28** | Anderson et al. (2017) | 4 NC (24) | 120 S (0) 32.9 | A history of psychosis, bipolar disorder, major depression, and anxiety disorders.  Covariates: N/A | AB: male inmates  P: PCL-R (I) | 1.5T fMRI; task ROI-A VBA | Implicit effect of emotional content: PCL-R total score negatively correlated with activity in within the visual stream including the right cuneus, left MOG, and left IOG. F1 negatively correlated with bilateral cuneus, left MOG, left IOG, and BA 18-19. F2 positively correlated with BA 25, bilateral amygdala, left IFG, left frontal midorbital, left frontal inferior orbital.  Explicit effect of emotional content: PCL-R total score (both F1 and F2) positively correlated with activity in the left vmPFC, left superior frontal cortex, left insula, left IOG, and left MOG. F2 showed a negative association with activity in the left amygdala.  Attention to emotion (during emotional pictures): F1 positively correlated with activity in the AG, left IOG, bilat. MOG.  Vigilance for emotion (during neutral slides): PCL-R total score negatively correlated with activity in the left MOG. F1 negatively correlated with activity in the bilateral cuneus, left IOG, left MOG. F2 positively correlated with activity in the amygdala bilaterally. |
| **29** | Jiang et al. (2017) | 2 ASPD v. HC | 32 ASPD (0) 20.5 35 HC (0) 21.67 | Previous mental disorder or behavioral disorder caused by brain damage  Covariates: age, IQ and education matched HC; | AB: youth offenders with ASPD; DSM-IV (I); PDQ (S) | 1.5T fMRI; Rest ROI-A VBA | ASPD vs. HC showed multiple disruptions in topological organization of functional brain networks. Decreased connectivity was identified in several locations and was mostly located between parietal and frontal gyri, or between these gyri and other brain regions denoting disruptions in the fronto-parietal control network. |
| **30** | Contreras-Rodríguez et al. (2014) | 4 P. (27.8) v HC (0.8) | 22 P (0) 39.8 22 HC (0) 40.6 | DSM-IV Axis 1 disorders (except for SUD), DSM-IV Axis 2 disorders (except for ASPD)  Covariates: age, normal IQ, and gender matched control group. | AB: male inmates convicted of violent crimes  P: PCL-R (I) | 1.5T fMRI; Task + rest ROI VBA | Brain response to emotional faces: P vs. HC showed increased activity in the right basal ganglia and right thalamus and decreased activation in right amygdala. P exhibited increased bilateral activation of visual cortex, medial frontal cortex, and the left PFC.   P showed a reduced functional connectivity between the left amygdala and bilateral visual cortices, the left fusiform gyrus, left parietal cortex, bilateral PFC, and the right thalamus.  F1 positively correlated with activity in the mPFC, right IFG, and left frontal operculum. F2 negatively correlated with left MFG, right IFG, left parietal lobe, right occipital lobe and right thalamus. |
| **31** | Hosking et al. (2017) | 4 NC (23.5) | 45 S (0) 31 | Age > 45, IQ < 70, history of psychosis, bipolar disorder, concussion or post-concussive syndrome, current psychotropic medication  Covariates: cumulative drug abuse score | AB: male offenders from two medium-security correctional facilities; SUD as per DSM-IV (I) and ASI (I);  P: PCL-R (I) | 1.5T fMRI; Task + rest ROI VBA | Total PCL-R score and F1 positively correlated with subjective value-related activity in the right NAcc.  PCL-R Total score (both F1 and F2) negatively correlated with functional connectivity between NAcc and vmPFC. |
| **32** | Vieira et al. (2014) | 1 P (90.61) v. C (50.88) | 18 P (44) 20.89 17 C (71) 21.24 | Neurological and psychiatric disorders including brain injuries  Covariates: subjects were divided to high (P) and low (C) psychopathy groups | P: TRiPM (S) | 3T fMRI; task Whole-brain VBA | Neural mechanisms in rejection of unfair offers: C showed increased activity in left dlPFC, whereas P showed increased activity in the right rostral ACC and vmPFC. |
| **33** | Fede et al. (2016) | 4 NC (20.73) | 245 S (0) 36.14 | English reading level below 4th grade, history of neurological disorder or stroke, head injury with loss of consciousness greater than 1 h, or history of psychotic disorder in the self or in a first-degree relative.  Covariates: N/A | AB: male inmates from two prisons  P: PCL-R (I) | 1.5T fMRI; task ROI-A VBA | Wrong vs not wrong moral stimuli: PCL-R total score negatively correlated with activity in the ACC.   Controversial moral stimuli: PCL-R total score negatively correlated with activity in the right TPJ, right dlPFC, and bilateral mPFC. |
| **34** | Han, Alders, Greening, Neufeld, & Mitchell (2012) | 1 P (79.19) v. C (24.25) | 16 P (63) 24.44 16 C (78) 25.25 | DSM-IV Axis I disorders or a history of neurological disorder  Covariates: Subject divided to high (P) and low (C) CU groups; age and IQ matched | P: PPI-R (S) | 3T fMRI; task Whole-brain and ROI VBA | Viewing expressions of fear eyes removed vs eyes only: C vs. P showed increased activity bilaterally in medial frontal gyrus, IPL, SFG, MFG, and right CG. Low CU traits correlated positively significantly greater left amygdala activity.   Viewing expressions of happiness eyes removed vs eyes only: C vs. P showed increased activity in the left fusiform gyrus, left MTG, and bilateral amygdala. |
| **35** | Vieira, Tavares, Marsh, & Mitchell (2017) | 1 NC (47.9) | 23 S (52) 20.96 | A history of psychiatric or neurological diagnoses, brain injuries or substance abuse.  Covariates: N/A | P: PPI-R (S) | 3T fMRI; task Whole-brain and ROI VBA | Main effect of approaching vs. receding images of emotional faces: increased activation bilaterally in insula, DLPFC, fusiform gyri, and visual cortices as well as right amygdala, left SPL, and right IPL. Amygdala activation was positively correlated with approaching images.   Activation of amygdala in response to sad faces negatively correlated with Coldheartedness. |
| **36** | Sadeh et al. (2013) | 1 NC | 49 S (61) 33.7 | Psychosis, mania, or current SUD  Covariates: N/A | P: NEO-FFI (S); DSM-IV (I) | 3T fMRI; task Whole-brain  VBA | Emotion-word Stroop test: Fearless-dominance factor moderated activation to positive vs neutral words mainly in left SMG/IPL, and right SFG. Impulsive-antisociality factor was associated with impaired attentional control in both positive vs. neutral and negative vs. neutral words. Impulsive-antisociality correlated positively with activity in left amygdala, left posterior/agranular OFC, and right agranular insula/OFC.   Fearless-dominance and impulsive-antisociality were associated with activity in the left MTG, left STG, left SMG, left precuneus, medial rostral ACC, medial OFC/frontal pole, and bilaterally in the pre- and postcentral gyri. |
| **37** | Sethi et al. (2015) | 4 P (28) v. HC (4) | 13 P (0) 40 HC 13 (0) 34 | English as first language, reading age of above ten years old, a history of significant head injury (i.e., leading to loss of consciousness for an hour or longer), neurological problems, DSM-IV Axis 1 disorders, or substance use disorder for the preceding month.  Covariates: age and IQ matched non-criminal HC | AB: male probationers convicted for at least one serious violent crime  P: PCL-R (I) | 1.5T DTI ROI-M DT | P vs. HC showed reduced FA in the left dorsal. Total PCL-R score and F1 negatively correlated with FA in the dorsal CG bilaterally. |
| **38** | Cope et al. (2012) | 4, 2 NC (18.4) | 66 S (45) 36-9 | Age < 18 or > 55, current pregnancy, English reading level lower than 4th grade, history of seizures or epilepsy or loss of consciousness > 30 min, psychotic disorder in a first degree relative.  Covariates: SUD, ASPD | AB: probationers, parolees, and persons who had sought treatment from drug treatment centers; ASI (I); DSM-IV (I)  P: PCL-R (I) | 3T MRI Whole-brain and ROI-M VBA | PCL-R total score negatively correlated with GMV in the left ITG, MTG, insula, uncus as well as right hippocampus, and lingual gyrus. PCL-R total score positively correlated bilaterally with SFG, MFG (parts of the dlPFC), caudate head, and thalamus as well as right gyrus rectus (part of OFC), precentral gyrus, ACC, and left caudate body.  F1 negatively correlated with GMV in the right ITG, left claustrum, and right CB. F1 positively correlated with GMV bilaterally in the SFG and precentral gyri as well as left MFG, gyrus rectus, caudate head, GP and putamen.  F2 negatively correlated with GMV the right medial frontal gyrus, insula, and caudate tail as well as left ITG, parahippocampal gyrus, fusiform gyrus, MTG, insula, and IPL. F2 positively correlated with GMV bilaterally in the SMG and MFG as well as left gyrus rectus, IFG, postcentral gyrus, caudate head, and right ACC. |
| **39** | Deeley et al. (2006) | 4 P (29.33) v. HC | 6 P (0) 36 9 HC (0) 27 | Current medication, comorbid psychiatric illness, neurological and extracerebral disorders that might affect brain function  Covariates: N/A | AB: male repeat violent crime offenders  P: PCL-R (I) | 1.5T fMRI; Task Whole-brain VBA | Facial expressions fear vs. neutral: P showed increased activity in the right insula and precuneus. P showed decreased activity in the right CB and fusiform gyrus.  Happy vs neutral: P showed increased activation the right CB, fusiform gyrus, MOG, ACC, and medial frontal gyrus as well as in the left SPL and bilateral precuneus. |
| **40** | Contreras-Rodríguez et al. (2015) | 4 P (27.8) v HC (0.8) | 22 P (0) 39.8 22 HC (0) 40.6 | DSM-IV Axis 1 disorders (except previous substance misuse), DSM-IV Axis 2 disorders (except ASPD)  Covariates: age, normal IQ, and gender matched HC | AB: male inmates convicted of violent crimes  P: PCL-R (I) | 1.5T fMRI; rest Whole-brain and ROI VBA | P vs. HC showed GMC and GMV reductions mainly in PFC, limbic-paralimbic structures. GMC reductions bilaterally in vmPFC, amygdala, hippocampus, insula-operculum as well as in the left MTG, ACC, in the right lateral frontal cortex, PCC, precuneus, and fusiform gyrus. F1 negatively correlated with GMC in bilateral amygdala, right dmPFC, insula-operculum, left parietal cortex, and CB. F2 positively correlated with GMC in bilateral lateral PFC, postcentral gyrus, temporal cortex, ventral frontal PFC, left insula-operculum, and dmPFC.   P showed GMV reductions in the right ventral PFC, amygdala, hippocampus, fusiform gyrus, in the left lateral frontal PFC, precuneus, and insula-operculum. F1 negatively correlated with GMV in bilateral amygdala, basal ganglia, lateral PFC, right dmPFC, insula-operculum, left temporal cortex, and CB. F2 positively correlated with GMV right dmPFC, lateral frontal PFC, CB, and left insula-operculum.   P showed increased functional connectivity in between medial-dorsal frontal cortices and dlPFC bilaterally. Reduced functional connectivity was shown between medial-dorsal frontal cortices, right anterior insula–frontal operculum, amygdalae and hypothalamus.   P showed decreased functional connectivity between amygdalae and medial and left frontal cortical areas.   F1 positively correlated with global functional connectivity in medial and lateral frontal areas |
| **41** | Müller et al. (2008) | 4 P (33.35) v. HC (0.53) | 17 P (0) 33.00 17 HC (0) 30.59 | DSM-IV Axis 1 disorders (except for SUD)  Covariates: SUD | AB: male criminals sentenced to psychiatric treatment  P: PCL-R (I) | 1.5T MRI ROI-A VBA | P showed decreased GMV in bilateral STG, right MFG, and right MCC. |
| **42** | Raine et al. (2004) | 1 SP (27.7) v. UP (31.5) v. C 10.9) | 12 SP (0) 29.5 16 UP (0) 33.8 23 C (0) 28.4 | All: Age < 21 or > 45, nonfluency in English, history of epilepsy  C: no criminal convictions  Covariates: subjects divided into successful (SP), unsuccessful (UP), and not psychopaths (C); Prior trauma exposure, head injury, schizophrenia-spectrum disorder | AB: NYS-AD (S); criminal history transcripts; DSM-IV (I);  P: PCL-R (I); IM-P (I); | 1.5T MRI ROI-M VBA | UP showed an abnormal asymmetry in the anterior hippocampi with the right side being larger relative to the left compared with SP + HC. |
| **43** | Sato el al. (2011) | 3 P (17.8) v. HC | 15 P (47) 32 15 HC (47) 32 | N/A  Covariates: Gender, age, and education matched HC without a history of neurological or psychiatric disorders or serious misconduct | AB: patients with ASPD; DSM-IV (I)  P: PCL-SV (I) | 1.5T MRI ROI-A VBA | P could be discriminated blindly from HC solely based on decreased GM in bilateral STS, right CG, and occipital peristriate cortex. |
| **44** | Tang, Jiang, Liao, Wang, & Luo (2013) | 2 ASPD v. HC | 32 ASPD (0) 20.50 34 HC (0) 21.67 | Previous mental disorder or behavioral disorder caused by brain damage  Covariates: age, IQ and education matched HC; reward | AB: youth offenders with ASPD; DSM-IV (I); PDQ (S) | 1.5T fMRI; rest Whole brain and ROI VBA | ASPD showed at multiple regions uncoupling in the default mode, attention and cerebellar networks at rest. ASPD could be discriminated from HC based on this altered functional connectivity particularly in regions of the left precuneus, the left SPG and the bilateral CB. Additionally, ASPD showed increased GMV in the right IPL and increased WMV in the right precuneus. |
| **45** | Korponay et al. (2017a) | 4 P (32.1) v. MP (25.6) v. C (15.3) | 41 P (0) 31.5 48 MP (0) 31.8 35 C (0) 31.3 | Age > 45, IQ < 70, history of psychosis, bipolar disorder, significant head injury or postconcussion symptoms, current psychotropic medication.  Covariates: SUD; subjects divided into high (P), medium (MP), and no psychopathy (C) | AB: male offenders from a medium-security correctional facility; DSM-IV (I)   P: PCL-R (I) | 1.5T MRI and fMRI; rest ROI VBA | Total PCL-R score positively correlated with GMV in bilateral NAcc and putamen. No significant correlations for F1. F2 positively correlated with GMV in NAcc bilaterally, in the right putamen, GP, and caudate.   Total PCL-R scores positively correlated with focal volume clusters bilaterally in the NAcc, GP, and in the left putamen. F1 negatively correlated with a focal volume cluster in the right putamen. F2 positively correlated with focal volume clusters bilaterally in NAcc, putamen, the right GP, and left caudate.  Total PCL-R scores negatively correlated with functional connectivity between the left putamen and right superior lateral occipital cortex; and between the right GP and right occipital cortex. No correlation for F1. F2 positively correlated between striatal seeds and the ventral midbrain, dlPFC, and other areas of the striatum. F2 negatively correlated between striatal seeds and the precentral gyrus, postcentral gyrus, and lateral occipital cortex. |
| **46** | Korponay et al. (2017b) | 4 P (32.1) v. MP (25.6) v. C (15.3) | 41 P (0) 31.5 48 MP (0) 31.8 35 C (0) 31.3 | Age > 55, IQ < 70, history of psychosis, bipolar disorder, significant head injury or postconcussion symptoms, current psychotropic medication.  Covariates: SUD; subjects divided into high (P), medium (MP), and no psychopathy (C) | AB: male offenders from a medium-security correctional facility; ASI (I)   P: PCL-R (I) | 1.5T MRI and fMRI; rest ROI VBA | Total PCL-R positively correlated with GMV in the right mOFC. No significant correlations for F1. F2 positively correlated with GMV in the left dlPFC (MFG and SFG) and right mOFC.  F2 positively correlated with focal GMV bilaterally in the dlPFC, mOFC, and right ACC. No significant correlations for PCL-R Total score or F1.   Total PCL-R score and F1 correlations for functional connectivity were not evaluated. F2 positively correlated with functional connectivity between left MFG and right anterolateral PFC as well as between right MFG and right FPC. |
| **47** | Sommer et al. (2010) | 4 P (28.6) v. C (9.6) | 14 P (0^[[1]](#footnote-1)^) 31.4 14 C (0) 29.2 | Neuropsychiatric diagnoses, neurological disorders, DSM-IV Axis 1 disorders (except SUD), anatomical brain abnormalities, and severe medical conditions; sex offenders; pedophilia.  Covariates: age, education level, and IQ matched control group; offenders divided to psychopaths (P) and nonpsycopaths (C). | AB: offenders with ASPD sentenced to psychiatric treatment; DSM-IV (I)  P: PCL-R (I) | 1.5T fMRI; Task Whole-brain VBA | Emotion attribution in cartoon stories with intension unfulfilled vs. intension fulfilled vs. non-mentalizing reality: C showed increased activity in bilateral SMG and the left SFG in all contrasts. P showed increased activity in the left STS in unfulfilled vs. reality contrast, increased activity in the left STS, IPL and precuneus in reality vs. fulfilled contrast, and increased activity in the AG in unfulfilled vs. fulfilled contrast.   P vs. C showed increased activity in the OFC, increased activity in mPFC in unfulfilled vs. reality contrast, and increased activity of the left TPJ in both unfulfilled and fulfilled vs. reality. |
| **48** | Glenn, Raine, Yaralian, &Yang (2010) | 1 P (27.2) v. C (12.9) | 22 P (9) 31.1 22 C (9) 31.0 | Age < 21 or > 45, nonfluency in English, history of epilepsy or psychosis  Covariates: age, gender, ethnicity, and SUD matched nonpsychopath control group; number of convictions | AB: criminal history transcripts  P: PCL-R (I) | 1.5T MRI ROI-M VBA | P vs. C showed a 9.6% increased total striatum volume attributed bilaterally to putamen and GP as well as right caudate body. PCL total score positively correlated with GMV in total striatum, in the right caudate body, left putamen and GP. F1 positively correlated with GMV in total striatum, in bilateral caudate bodies, and left putamen and GP. F2 correlated positively GMV in total striatum and bilaterally in putamen and GP. |
| **49** | Wolf et al. (2015) | 4 P (31.90) v. MP (25.54) v. C (14.41) | 50 P (0) 21.84 47 MP (0) 30.97 50 C (0) 21.26 | Age < 18 or > 45, IQ < 70, a history of psychosis or bipolar disorder or loss of consciousness > 30 min, current psychotropic medication  Covariates: subjects divided to age, IQ and education level matched high (P), medium (MP) and nonpsychopath groups (C); SUD; | AB: male offenders from a medium-security correctional facility; DSM-IV (I)  P: PCL-R (I) | 1.5T DTI ROI-A TBSS | P vs. C showed decreased FA in the right UF. PCL-R Total score and F1 negatively correlated with FA in the right UF. No correlations found for other WMTs. |
| **50** | Juárez, Kiehl, & Calhoun (2013) | 4 P (32.5) v. MP (24.5) v. C (15.6) | 17 P (0) 32.1 37 MP (0) 34.3 48 (0) 35.8 | N/A  Covariates: subjects divided into high (P), medium (MP), and low (C) psychopaths; SUD | AB: male offenders from prisons  P: PCL (I) | 1.5T fMRI; task ROI-A VBA | Psychopathy was associated with functional connectivity changes in the default mode, frontoparietal, and visual/PCC networks.   Total PCL-R score, F1 and F2 negatively correlated with changes in the frontoparietal and visual/PCC networks. Total PCL-R score, F1 and F2 positively correlated with changes in the DMN. |
| **51** | Ermer, Cope, Prashanth, Calhoun, & Kiehl (2012) | 4 NC (21.3) | 254 S (0) 33.9 | A history of psychotic or bipolar disorders, traumatic brain injury with loss of consciousness > 1h  Covariates: SUD | AB: male offenders from medium and maximum security correctional facilities; DSM-IV (I); ASI (I)  P: PCL-R (I) | 1.5T MRI Whole-brain and ROI-A VBA | Psychopaths had decreased GMV and GMC in several paralimbic and limbic areas bilaterally: parahippocampal gyri, amygdala, hippocampi, PCC and OFC. Decreased GMV was also found in temporal poles and inferior temporal cortices.  Total PCL-R score negatively correlated with GMV in the left lateral OFC, bilateral temporal pole, right hippocampal gyrus, right amygdala, right hippocampus, and PCC.  Total PCL-R score negatively correlated with GMC in hippocampi, parahippocampal gyri, amygdalae, bilateral temporal pole, and left lateral OFC.  F2 was negatively associated with the right temporal pole GMV. No other associations found for F1 and F2. |
| **52** | Baskin-Sommers, Neumann, Cope, & Kiehl (2016) | 4 NC (21.3) | 254 S (0) 33.9 | A history of psychotic or bipolar disorders, traumatic brain injury with loss of consciousness > 1h  Covariates: SUD, age, IQ | AB: male offenders from medium and maximum security correctional facilities; DSM-IV (I); ASI (I)  P: PCL-R (I) | 1.5T MRI ROI-A VBA | Structural equation modeling showed that PCL-R superordinate psychopathy is in particular associated with decreased GMV in the limbic/paralimbic system.   F1 negatively correlated with VBM latent factors in the right parahippocampus, hippocampus, and amygdala. F2 negatively correlated with VBM latent factors in the left corresponding structures. |
| **53** | Miskovich et al. (2018) | 4 NC (22.02) | 716 S (0) 31.8 | Age < 18 or > 45, IQ > 70, a history of schizophrenia or schizoaffective disorder or neurological disorders traumatic brain injury with loss of consciousness > 1h  Covariates: SUD, age, IQ | AB: male offenders from medium and high security correctional facilities; DSM-IV (I); ASI (I);  P: PCL-R (I) | 1.5T MRI ROI-A VBA | Total PCL-R score negatively correlated with gyrification in the right MCC extending into dmPFC, and right lateral superior parietal cortex. F1 negatively correlated with gyrification in the right MCC. |
| **54** | Philippi et al. (2015) | 4 P (31.1) v. C (14.8) | 46 P (0) 29.6 49 C (0) 30.4 | Age > 45, IQ < 70, history of psychosis, bipolar disorder, significant head injury or postconcussion symptoms, current psychotropic medication  Covariates: subjects divided into psychopathic (P) and nonpsycopathic groups (C) | AB: male offenders from a medium-security correctional facility  P: PCL-R (I) | 1.5T fMRI; rest ROI VBA | P vs. C showed reduced functional connectivity between the right IPS and right precuneus; between the right IPS, bilateral dACC and right insula in the frontoparietal network (FPN).   Total PCL-R scores positively correlated with reduced connectivity between the right IPS, bilateral dACC and right precuneus. No associations found for cingulo-opercular or default mode networks in between-group analysis or regression analyzes with total PCL-R score.  F1 positively correlated with reduced connectivity between the medial and lateral DMN regions: between PCC and right SFG; between left IPL and left SFG; and between right IPL and left PCC.  F2 positively correlated with increased connectivity between the medial prefrontal and parietal DMN regions: between mPFC and left dmPFC, left SFG, and right PCC; between PCC and left dmPFC, left SFG and left MFG. In the FPN, F2 positively correlated with increased connectivity between the mPFC and left precuneus. |
| **55** | Müller et al. (2003) | 4 P (36.8) v. HC | 6 P (0) 33.0 6 HC (0) 28.0 | Neuropsychiatric disorders other than psychopathy personality disorder, history of serious head trauma, IQ < 85  Covariates: N/A | AB: Criminal male psychopaths from a high-security psychiatric facility  P: PCL-R (I) | 1.5T fMRI; task Whole-brain and ROI VBA | Pictures with negative emotional content: P vs. HC showed increased bilateral activation of the MTG, occipital and parietal cortices as well as left precentral cortex, STG, right IFG, medial frontal gyrus, ACC, and amygdala. P vs. HC showed decreased activation of the right subgenual cingulate, MTG, fusiform gyrus, left lobulus paracentralis, dACC, and parahippocampal gyrus.  Pictures with positive emotional content: P vs. HC showed increased activation of the fusiform cortices, parietal cortices, cerebellar hemispheres, temporal and precentral cortices, and left IFG. P vs. HC showed decreased activation of the bilateral occipital cortex, right medial frontal gyrus and right MTG. |
| **56** | Harenski, Harenski, Shane, & Kiehl (2010) | 4 P (31.8) v. C (13.3) | 16 P (0) 33.3 16 C (0) 34.8 | Age < 18 or > 55non-fluency in English, reading level lower than 4th grade, IQ < 80, history of seizures, prior head trauma with loss of consciousness > 30 min, Axis 1 diagnosis, lifetime history of psychotic disorder, psychotic disorder in a first degree relative, current alcohol or drug use.  Covariates: subjects divided into psychopathic (P) and nonpsycopathic groups (C) ; SUD | AB: male offenders from a medium-security correctional facility P: PCL-R (I) | 1.5T fMRI; Task ROI-A VBA | C vs. P showed a significantly increased activity in the anterior temporal cortex (BA 21) and vmPFC (BA 10) during moral vs. to non-moral and neutral picture distinction. In C, moral violation severity ratings and were associated with increased amygdala activity. In P, moral violation severity ratings were associated with increased right posterior temporal cortex (BA 39) activity.   F1 negatively correlated with activity in the right amygdala. F2 negatively correlated with vmPFC activity. |
| **57** | Mier et al. (2014) | 4 P (26.67) v. HC (326.53) | 11 P (0) 44.55 18 HC (0) 44.00 | N/A  Covariates: age, education level, and IQ matched control group; | AB: male offenders sentenced to psychiatric treatment for violent crimes P: PCL-R (I); PPI-R (S) (C only) | 3T fMRI; task Whole-brain and ROI VBA | The pattern of activation with recognizing emotions and intentions was found in bilateral STS, inferior prefrontal gyrus, and in bilateral amygdala in HC. This pattern was not activated in P. P vs. HC showed hypoactivation of the right fusiform gyrus when processing facial emotional information. HC showed increased activation in the bilateral STS, bilateral BA 44 and left amygdala, with the concept of putting oneself into the shoes of another person. This activation was not detected in P. Furthermore, P showed decreased connectivity between right STS and left amygdala. |
| **58** | Kiehl et al. (2001) | 4 P (32.8) v. C (16.6) v. HC | 8 P (0) 33.9 8 C (0) 37.1 8 HC (0) 31.9 | A history of serious head injury with loss of consciousness for more than 1 hour, psychotic illness in self or first-degree relative, non-native in English, DSM-IV criteria for SUD within the last 6 months  Covariates: inmates divided into psychopaths (P) and nonpsychopaths (C);  age, parental socioeconomic status, IQ, and gender matched groups. | AB: male inmates from a maximum security prison  P: PCL-R (I) | 1.5T fMRI; task ROI VBA | During affective memory task, P showed decreased activity in the rostral and caudal ACC, PCC, left IFG, right amygdala, and VS compared to C + HC. P showed decreased activity in left amygdala, parahippocampal gyrus, bilateral anterior STG compared to HC. |
| **59** | Yang, Raine, Narr, Colleti, & Toga (2009) | 1 P (27.96) v. C (10.56) | 27 P (7) 32.22 32 C (19) 30.84 | N/A  Covariates: SUD | AB: NYS-AD (S); criminal history transcripts; DSM-IV (I);  P: PCL-R (I) | 1.5T MRI ROI VBA | P showed a 17.14% volume reduction in the left amygdala and an 18.93% volume reduction in the right amygdala compared to C.   Total PCL-R score negatively correlated with amygdalae volumes. F1 negatively correlated with amygdalae volumes. F2 negatively correlated with right amygdala volume. |
| **60** | Pardini, Raine, Erickson, & Loeber (2014) | 1, 2 P (143.85) v. MP (137.50) v. C (118.31) | 20 P (0) 26.55 6 MP (0) 26.53 20 C (0) 26.76 | A history of psychotic disorder, neurological disease, structural brain injury, postconcussive syndrome, or cardiovascular disease, current use of psychotropic medications, IQ below 70, current incarceration  Covariates: subjects from longitudinal cohort study divided to three groups:self-reported or charged with violence across > 4 years (P), self-reported or charged with violence between 1 and 3 years (MP), and no history of serious violence (C); age, IQ, and race matched groups | AB:  Adolescence (mean age 16 years): RPQ (S); YSR (S); SRD (S) Adulthood (concurrent with neuroimaging; mean age 26 years): ASR (S); AQ-SF (S); IPAS (S); ASRS (S); SRD (S); VHQ (S) Postscan (mean age 29 years): criminal history transcripts; AQ-SF (S); SRD (S)  P:  Childhood (mean age 7.5-11 years): TRF (T)  Adolescence (mean age 16 years): CPS-R (S) Adulthood (concurrent with neuroimaging; mean age 26 years): SRP-III (S) Postscan (mean age 29 years): SRP-III (S) | 3T MRI ROI-A VBA | Decreased bilateral amygdala volume was associated with higher levels of psychopathy from childhood to adulthood, and successfully predicted increased psychopathic features and committing violent acts in a 3-year follow up.   Left amygdala volume negatively correlated with the lifestyle dimension of psychopathy. Right amygdala volume negatively correlated with the interpersonal and affective dimensions. |
| **61** | Kumari et al. (2014) | 4 ASPD v. HC | 14 ASPD (0) 36.64 15 HC (0) 32.13 | All: age < 18 or > 55, current substance abuse, history of neurological conditions or head injury, nonfluency in English  HC: history of a mental disorder or violence  Covariates: age, gender, and IQ matched HC; psychosocial deprivation | AB: male ASPD inmates from medium and high security psychiatric hospitals; The Gunn and Robertson criminal profile (I) | 1.5T MRI ROI VBA | ASPD showed lower ACC volume compared to HC. ACC volumes correlated negatively with total psychosocial deprivation. |
| **62** | Yang, Raine, Colletti, Toga, & Narr (2010) | 1 UP (30.06) v. SP (25.10) v. HC (10.44) | 16 UP (6) 33.62 10 SP (10) 30.20 27 HC (22) 30.11 | N/A  Covariates: psychopaths were further classified into unsuccessful (UP) and successful psychopaths (SP); SUD and socioeconomic status | AB: NYS-AD (S); criminal history transcripts; DSM-IV (I);  P: PCL-R (I); IM-P (I); | 1.5T MRI ROI-M VBA | UP showed GMV reductions in the right MFC right, bilateral OFC, and the right RG compared with C. UP vs. SP showed GMV reductions bilaterally in OFC.  UP vs. C showed cortical thinning in the right MFC, bilateral OFC, temporal cortex, and PCC. SP showed no significant overall cortical thinning effects compared with C.  UP showed GMV reductions in the right (20%) and left (26%) amygdala along with abnormal morphology compared with C. SP showed GMV reductions in the right (9.3%) and left (12.7%) amygdala compared with C without abnormal morphology. No significant findings in SP vs. UP. |
| **63** | Geurts et al. (2016) | 4 P (30.6; 362.2) v. C1 (323.7) v. C2 (368.3) | 14 P 40.1 10 C1 42.5 10 C2 39.1 | All: DSM Axis 1 and 2 disorders, current psychotropic medication (except for oxazepam)  C: criminal record  Covariates: controls divided into groups of low (C1) and high impulsive/antisocial traits and (C2); all groups age and IQ matched | AB: offenders from a high security forensic psychiatric hospital  P: PCL-R (I) (not for C); PPI-R (S) | 3T fMRI; task ROI VBA | Compared to C1, C2 and P showed increased reward expectancy related activity in VS. No differences in activity between C2 and P. P showed increased functional connectivity between VS and dmPFC compared to C2. No correlation found for PCL-factors.  Supplementary post hoc analysis with groups divided by fearless/dominance traits: P showed decreased reward expectancy related activity in PAG compared to non-criminals who also score high on fearless/dominance traits.. |
| **64** | Harenski, Edwards, Harenski, & Kiehl (2014) | 4 P (18.7) v. HC | 157 P (100) 33.2 46 HC (100) 27.0 | All: age < 18 or > 50, reading level below 4th grade, IQ < 75, a history of seizures, DSM-IV Axis 1 diagnosis apart from SUD, history of a psychotic disorder in self or first degree relative.  HC: a history of SUD  Covariates: SUD | AB: female inmates from a medium security correctional facility.   P: PCL-R (I) | 1.5T fMRI; task Whole-brain and ROI VBA | Main effect moral vs. non-moral pictures: increased activity in the vmPFC, TPJ and PCC in both groups. Total PCL-R score negatively correlated with activity in the right parahippocampal gyrus, fusiform gyrus, and TPJ. F1 negatively correlated with activity in the right parahippocampal gyrus.  Main effect of moral + non-moral vs. non-moral pictures: increased activity in the amygdala, mPFC, ACC and vlPFC in both groups. F2 negatively correlated with activity in the right amygdala, and rostral ACC. |
| **65** | Zijlmans et al. (2018) | 3 P (34.08) v. HC 35.50) | 100 P (0) 22.56 22 HC (23.19) | Non-corrected deficient vision  Covariates: cannabis use; age and gender matched HC | AB: multiproblem young adults from a social welfare agency  P: YPI-S (S) | 3T fMRI; task Whole-brain and ROI VBA | Main effect of moral vs. non-moral pictures: increased activity the right STG, vmPFC, precuneus, parahippocampal gyrus, CB, and in the left MOG in both groups.   Immoral vs nonmoral images: total YPI score positively correlated with activity in the left vmPFC and left STG. CU factor positively correlated with activity in the left vmPFC, CG and STG. No other associations were found. |
| **66** | Pujara, Motzkin, Newman, Kiehl, & Koenigs (2014) | 4 P (31.7) v. C (14.1) | 18 P (0) 32.2 23 C (0) 32.4 | Age > 45, IQ < 70, history of psychosis, bipolar disorder, significant head injury or postconcussion symptoms, current psychotropic medication  Covariates: subjects divided into psychopathic (S) and nonpsycopathic groups (C) ; SUD | AB: male offenders from a medium-security correctional facility  P: PCL-R (I) | 1.5T fMRI; task ROI VBA | Main effect of gain vs. loss: increased activation of left ventral striatum in both groups. Total PCL-R score correlated positively with left VS activity.   Loss v. neutral: main effect in both groups was decreased activation of left ventral striatum. Total PCL-R score negatively correlated with activity in the left VS.  Furthermore, PCL-R total score positively correlated with right NAcc volume in P, but not in C. No other associations found. |
| **67** | Rilling et al. (2007) | 1 NC | 30 S (50) 21.1 | N/A  Covariates: N/A | P: PPI-S (S); LP (S) | 3T fMRI; task ROI-M VBA | Main effect of time choosing in-between to cooperate or betray in Prisoner’s Dilemma: decreased activity in rostral anterior ACC and dlPFC in whole group. Psychopathy scores correlated negatively with activity in the OFC and positively with dlPFC.  Total psychopathy scores positively correlated with both the number of times a player betrayed as well as with the probability of betrayal after a mutually benefitting interaction in the previous round of the game.  Higher psychopathic traits were associated with reduced amygdala activity when the cooperation efforts in Prisoner’s Dilemma were not reciprocated. |
| **68** | Schiffer et al. (2017) | 4 ASPD (12.3) v. HC (4.4) | 18 ASPD (0) 35.3 18 HC (0) 36.3 | N/A  Covariates: SUD | AB: violent male offenders from forensic hospitals and prisons; ASPD as per DSM-IV (I);   P: PCL-SV (I); IRI (S); | 1.5T fMRI; task ROI-A VBA | Recognizing emotions based on eyes: No performance differences between ASPD and HC. ASPD showed increased activations in the left mPFC, left vlPFC, left pSTS/TPJ, left fusiform gyrus, and left precuneus compared to HC. ASPD showed decreased activation of left amygdala compared to HC. (68) |
| **69** | Schiffer et al. (2014) | 4 ASPD (11.9) v. HC (5.1) | 21 ASPD (0) 35.2 23 HC (0) 34.1 | All: Axis 1 disorders (except SUD); a history of serious medical or neurologic illness, head injury, or color blindness.  HC: A history of violent behavior leading or not leading to prosecution  Covariates: age, gender, level of education, verbal IQ, and SUD matched HC | AB: violent male offenders from forensic hospitals and prisons; ASPD as per DSM-IV (I); BIS-11 (S);  P: PCL-SV (I); | 1.5T fMRI; task ROI-A VBA | Stroop test conflict related activity: ASPD showed decreased activity in the left dACC, and the left superior temporal cortex (including Wernicke’s area), but not in the dlPFC compared to HC. The left amygdala was the only region which was more activate in ASPD. F1 negatively correlated with dACC activity in both groups.  Error-related activity: ASPD showed decreased activity in right postcentral gyrus and precentral gyrus as well as in the left thalamus (ventral anterior nucleus), putamen, ACC, left MFG (dlPFC). |
| **70** | Yoder, Harenski, Kiehl, & Decety (2015) | 4 P (31.9) v. C (15.8) | 28 P (0) 32.6 34 C (0) 30.1 | Age >50, IQ < 70, a history of bipolar or major depressive disorder  Covariates: subjects divided into groups of high (P) and low (C) psychopathy; age and IQ matched | AB: male offenders from medium security correctional facilities  P: PCL-R (I) | 1.5T fMRI; task ROI-A VBA | Implicit moral evaluation of scenes depicting interpersonal harm or interpersonal assistance: Total PCL-R score and F2 negatively correlated with activity in the right caudate, dlPFC, and left precentral gyrus and fusiform gyrus. F1 negatively correlated with activity in the right dlPFC, left precentral gyrus and fusiform gyrus. Total PCL-R score and F1 positively correlated with activity in the right temporal pole, subgenual ACC, and left AIC. F2 positively correlated with activity in the right temporal pole and left insula.   Explicit moral evaluation: Total PCL-R scores negatively correlated with activity in the brainstem, parahippocampal gyri extending to left amygdala, pons, left CB, AG, postcentral gyrus, dACC and superior parietal cortex, and right lingual gyrus. F1 negatively correlated with activity in the brainstem, pons, and left parahippocampal gyrus. F2 had the same negative correlations as total PCL-R except for brainstem. Total PCL-R score as well as F1 and F2 positively correlated with activity in the left putamen, right thalamus, and left SFG.   Total PCL-R score negatively correlated with functional connectivity between the right amygdala and vmPFC; and between rTPJ and dACC in the implicit condition. Total PCL-R score negatively correlated with functional connectivity between rTPJ, right temporal pole, IFG, dmPFC and AIC; and positively between rTPJ and dACC in the explicit condition. In both conditions, total PCL-R score negatively correlated with functional connectivity between amygdala and caudate/putamen. |
| **71** | Decety, Skelly, Yoder, & Kiehl (2014) | 4 P (≥30) v. MP (21-29) v. C (≤ 20) | 27 P (0) 25 MP (0) 28 C (0) | Age < 18 or > 50  Covariates: subjects divided to groups of high (P), medium (MP), and low psychopathy (C);  age, IQ, ethnicity, and DSM-IV Axis II comorbidity matched. | AB: adult male offenders from medium security correctional facilities; DSM-IV (I)  P: PCL-R (I) | 1.5T fMRI; task ROI-A VBA | Expressions of happiness: C vs. P showed increased activity bilaterally in fusiform gyrus, IFG, dmPFC, inferior temporal pole, MTG, SMA, and in the right vmPFC. P vs. C showed increased activity in the right amygdala and bilateral superior temporal pole. Both F1 and F2 negatively correlated with activity in the bilateral fusiform gyrus, in the right mOFC, dmPFC, IFG, and left inferior temporal pole. F1 negatively correlated with activity in the bilateral IFG, right MOG, vmPFC, inferior temporal pole, left mOFC and dmPFC. F2 negatively correlated with activity in the right SMG and SMA. Expressions of fear: C vs. P showed increased activity bilaterally in fusiform gyrus, MOG, insula, IFG, SMA, MFG, in the right amygdala and vmPFC. P vs. C showed increased activity in the right anterior insula and left superior temporal pole.  Both F1 and F2 negatively correlated with activity in the bilateral MOG, right IFG and SMG. F1 negatively correlated with activity in the left insula and OFC, and in the right vmPFC and SMA. F2 negatively correlated with activity in the right insula, in the left IFG, MTG and SMA.   Expressions of sadness: C vs. P showed increased activity bilaterally in the IFG, amygdala, dmPFC, in the left fusiform gyrus, pSTS, in the right vmPFC, MFG and SMA. P vs. C showed increased activity in the left anterior insula and MCC.  Both F1 and F2 negatively correlated with activity in bilateral dmPFC, left pSTS, right IFG and SMA. F1 negatively correlated with activity in the left IFG, in the right vmPFC and MFG. F1 positively correlated with activity in the left anterior insula and MCC. F2 negatively correlated with activity in the left fusiform gyrus and inferior temporal pole.   Expressions of pain: C vs. P showed increased activity bilaterally in the IFG, MCC, AG, putamen, pSTS, SMG, dmPFC, mOFC, vmPFC, and dACC. P vs. C showed increased activity bilaterally in the anterior insula, postcentral gyrus, IPL, precentral gyrus, and in the right amygdala. Both F1 and F2 negatively correlated with activity in bilateral IFG, mOFC, dmPFC, MCC, AG, and right MFG. Both F1 and F2 positively correlated with activity in the left anterior insula. F1 negatively correlated with activity in left pSTS and right AG. F1 positively correlated with right anterior insula, precentral gyrus, and left postcentral gyrus, and left MCC. F2 negatively correlated with activity in right pSTS, dACC, and striatum. |
| **72** | Seara-Cardoso, Viding, Lickley, & Sebastian (2015) | 1 NC (29.5) | 46 S (0) 27.93 | N/A  Covariates: IQ and trait anxiety | P: SRP-SF (S) | 1.5T fMRI; task Whole-brain and ROI-A VBA | Main effects pain vs. no-pain emotional images: increased activity in left middle temporal gyrus, right occipital gyrus, bilateral supramarginal gyrus, left precentral gyrus, right cerebellum, right inferior frontal gyrus, left insula, right precentral gyrus, left inferior frontal gyrus, left postcentral gyrus, and right amygdala in whole group.   Affective-interpersonal traits negatively correlated with activity bilaterally in anterior insula, IFG, and MCC. Lifestyle-antisocial traits positively correlated with activity bilaterally in anterior insula, IFG, and MCC. |
| **73** | Glenn, Yang, Raine, & Colletti (2010) | 1 P (28.0) v. C (11.5) | 24 P (0) 32.7 24 C (0) 29.2 | Nonfluency in English, a history of epilepsy  Covariates: subjects divided into psychopaths (P) and nonpsychopaths (C) | AB: NYS-AD (S); criminal history transcripts; DSM-IV (I);  P: PCL-R (I); IM-P (I); | 1.5T MRI ROI-M VBA | No differences between the two groups on volume of the total ACC. |
| **74** | Walters, Ermer, Knight, & Kiehl (2015) | 4 NC (21.3) | 254 S (0) 33.63 | A history of psychotic or bipolar disorders, traumatic brain injury with loss of consciousness > 1h, missing data.  Covariates: SUD | AB: male offenders from medium and high security correctional facilities; SUD DSM-IV (I) and ASI (I);  P: PCL-R (I) | 1.5T MRI Whole-brain and ROI-A VBA | The structure of psychopathy is a quantitative continuum rather than categorical in nature as measured by taxometric analyses of PCL-R and GMC indicators of paralimbic system dysfunction |
| **75** | Laakso et al. (2002) | 4 P (27.6) v. HC | 24 P (0) 31 33 HC (0) 34 | Previous mental disorder or behavioral disorder caused by brain damage.  Covariates: Cloninger type 2 alcoholism, polysubstance abuse; age matched HC | AB: persons with ASPD on pretrial forensic evaluation and charged with violent offences; DSM-IV (I); ICD-10 (I)  P: PCL-R (I) | 1.0T or 1.5T MRI ROI-A VBA | P vs. HC showed decreased GMV in the left dlPFC, OFC, and MFG compared to HC. No associations found for PCL-R. |
| **76** | Bjork, Chen, & Hommer (2012) | 1 NC (368) | 31 S (42) 31.0 | A history of psychiatric, or neurological diagnoses, significant medical illness, SUD.  Covariates: N/A | AB: DSM-IV (I)  P: PPI-R (S) | 3T fMRI; task ROI VBA | Monetary reward anticipation positively correlated with activity in ventral striatum and anterior mesofrontal cortex (medial prefrontal cortex). PPI total score positively correlated with activity in ventral striatum, anterior mesofrontal cortex, and the right NAcc. Factors were not calculated for correlations. |
| **77** | Fullam, McKie, & Dolan (2009) | 1 NC (364.71) | 24 S (0) 30.04 | N/A  Covariates: subjects recruited from university rugby teams | P: PPI-R (S) | 1.5T fMRI; Task ROI VBA | Main effect of lying vs. truth: increased activity in bilateral vlPFC.   Fearlessness negatively correlated with activity in the right OFC. Coldheartedness negatively correlated with activity in bilateral temporal poles. Stress immunity negatively correlated with activity in bilateral insula. Machiavellian egocentricity negatively correlated with activity in bilateral caudate. No associations found for PPI total score. |
| **78** | Vieira et al. (2015) | 1 NC (71.31) | 35 S (0) 21.06 | Neurological and psychiatric disorders including brain injuries.  Covariates: subjects divided to high (S) and low (C) psychopathy groups. | P: TRiPM (S); PPI-R (S) | 3T MRI Whole-brain and ROI VBA | TRiPM total score negatively correlated with GMV in left putamen and left amygdala. TRiPM total score positively correlated with GMV in left caudate and left lateral OFC.  Boldness positively correlated with GMV in the right insula and pons. Meanness negative correlated with GMV in the left amygdala, and positively with left lateral OFC and left striatum. Disinhibition negatively correlated with GMV in the left amygdala. |
| **79** | Anderson, Maurer, Steele, & Kiehl (2018) | 4 NC (21.3) | 168 S (0) 37.1 | IQ < 70, diagnosis of schizophrenia, bipolar, current major depression, anxiety disorder, or ADHD; a history of serious and/or repeated head injury resulting in prolonged loss of consciousness.  Covariates: SUD | AB: male inmates from medium security correctional facilities  P: PCL-R (I) | 1.5T fMRI; task Whole-brain and ROI-A VBA | Psychopathy was associated with widespread disruption across the default mode and salience networks. F1 negatively correlated with activity bilaterally in anterior temporal cortex, mPFC, dACC, TPJ, PCC, amygdala, parahippocampal gyrus, insula and CB. |
| **80** | Pape et al. (2015) | 1, 2 NC (83.2) | 145 S (14) 17.6 | N/A  Covariates: age, IQ, and gender | AB: persons arrested before the age of 12  P: YPI (S) | 3T DTI Whole-brain TBSS | Grandiose-manipulative traits positively correlated with FA bilaterally in ATR, CT, Fmin, IFOF, UF, and CC. Grandiose-manipulative traits negatively correlated with RD bilaterally in ATR, CT, CG, Fmaj, Fmin, IFOF, ILF, SLF, UF, TSLF, and CC. CU traits positively correlated with AD in bilateral CT. |
| **81** | Ewbank et al. (2018) | 1 ECD (120) v. ACD (115) v. HC (95) | ECD 24 (0) 18.1 ACD 22 (0) 17.6 HC 25 (0) 18.3 | IQ < 85, the presence of a pervasive developmental disorder or chronic physical illness  Covariates: subjects divided to adolescence-onset (ACD) and childhood-onset (ECD) CD groups; age, IQ and ethnicity matched groups; ADHD | AB: persons from Pupil Referral Units and Youth Offending Services; K-SADS (I)  P: YPI (S) | 3T fMRI; task ROI VBA | Main effect angry vs. neutral faces: all groups showed increased activity in bilateral amygdala. ECD + ACD vs. HC showed reduced activity in the right amygdala. No differences between ECD and ACD. No correlations between overall psychopathic traits and amygdala responses. All groups showed reduced connectivity between vACC and bilateral amygdala. ACD + ECD and ACD alone showed a negative correlation between overall psychopathic traits and functional connectivity between vACC and left amygdala.  Main effect sad vs. neutral faces: all groups showed increased activity in the left amygdala. ECD + ACD vs. HC showed reduced activity in bilateral amygdala. Also, ECD vs. HC showed reduced activity in bilateral amygdala. ACD vs. HC no correlations. No correlations between overall psychopathic traits and amygdala responses.  All groups showed reduced connectivity between vACC and left amygdala. No correlation with psychopathic traits. |
| **82** | Deming et al. (2018) | 4 NC (22.9) | 57 S (0) | Age < 18 or > 55, a history of psychosis, bipolar disorder, epilepsy, stroke, or head injury with a loss of consciousness > 30 min; current psychotropic medication, English reading level below 4th grade, normal visual and auditory capabilities, IQ < 70  Covariates: SUD | AB: male inmates from a medium security correctional facility; DSM-IV (I)  P: PCL-R (I) | 1.5T fMRI; task Whole -brain and ROI-M VBA | Self-focused vs. other-focused judgment main effect: increased activity bilaterally in mPFC, PCC, precuneus, IFG, and anterolateral temporal cortex. Reduced activity bilaterally in dlPFC and lateral parietal cortex.   No correlations found for PCL-R F1. F2 negatively correlated with activity in right and left PCC, and right TPJ. |
| **83** | Cope et al. (2014) | 4 NC (20.17) | 137 S (68) 34.03 | IQ < 70, reading level below 6th grade, current antipsychotic medication, psychotic disorder in self or a first-degree relative, past or current central nervous system disease.  Covariates: SUD | AB: inmates from a two correctional facilities; DSM-IV (I); ASI (I)  P: PCL-R (I) | 1.5T fMRI; task ROI-A VBA | Main effect of viewing drug-related vs neutral images: increased activity bilaterally in insula, hippocampus, amygdala, caudate, putamen, NAcc, thalamus, ACC, and PCC.   PCL-R total score negatively correlated with activity in the right SFG, IFG, precuneus, putamen, in the left MFG, ACC, mid-CG, and bilateral GP. F1 positively correlated with activity in the right precentral gyrus, MOG, in the left insula, lateral GP, and bilateral culmen (CB). F2 negatively correlated with activity bilaterally in the SFG, MFG, CG, IPL, claustrum (CB), postcentral gyrus, in the left medial frontal gyrus, precentral gyrus, amygdala, ACC, transverse temporal gyrus, insula, MOG, cuneus, lateral and medial GP, left caudate tail, in the right IFG, parahippocampal gyrus, hippocampus, PCC, STS, MTG, and caudate body and head. |
| **84** | Sobhani, Baker, Martins, Tuvblad, & Aziz-Zadeh (2015) | 1, 2 NC (287.5) | 24 S (0) 18.9 | Non-corrected deficient vision; a history of neurological or psychiatric disorders.  Covariates: subjects recruited from a longitudinal study of antisocial and behavior in childhood; age and IQ | P: Concurrent: PPI (C) from prior (age 14-15) CPS (S), PCL-YV (I), APSD (S) | 3T DTI ROI-M DT | PPI score negatively correlated with FA in the right UF. |
| **85** | de Oliveira-Souza et al. (2008) | 3 P (17.8) v. HC (0.4) | 15 P (47) 32 15 HC (47) 32 | A history of chronic systemic diseases, head trauma with loss of consciousness; past or current illness associated with psychosis.  Covariates: age, gender and educational level matched HC | AB: patients with ASPD; DSM-IV (I)  P: PCL-SV (I) | 1.5T MRI Whole-brain and ROI VBA | P vs. HC showed GMV reductions bilaterally in the STS, insula, in the left mOFC, FPC, and anterior temporal cortex.   PCL-SV total score and F1 negatively correlated with GMV in mOFC extending to subgenual cingulate, frontopolar cortices, and STS. No correlations for F2. |
| **86** | Lam et al. (2017) | 3, 4 NC (30.04) & V v. NV | 67 (16) 34.09 23 V (17) 36.91 44 NV (16) 32.61 | N/A  Covariates: subjects divided into violent (V) and non-violent (NV) groups; schizophrenia | AB: inmates from a psychiatric hospital; DSM-IV (I)  P: PCL-R (I) | 1.5T MRI ROI VBA | F1 positively predicted violence and correlated negatively with GMV in the left caudate, bilateral putamen, and right lateral OFC. No correlations found for PCL total score or F2. |
| **87** | Gregory et al. (2015) | 2 P (28.2) v. ASPD (15.9) v. HC (3.4) | 12 P (0) 40.1 20 ASPD (0) 36.8 18 HC (0) 34.8 | DSM-IV Axis I disorders or self-reported neurological disorder, head injury resulting in loss of consciousness > 1h, severe visual och hearing impairments, reading age below 10 years  Covariates: ASPD males was divided to psychopaths (P) and nonpsychopaths (ASPD); IQ matched ASPD and P. | AB: male probationers with ASPD convicted for violent crimes; DSM-IV (I)  P: PCL -R (I) | 1.5T fMRI; task ROI | Punished errors vs. reward: P vs. ASPD showed increased activity bilaterally in PCC, precuneus, and in the right anterior insula. P vs. HC showed increased activity bilaterally in PCC and precuneus.   Total PCL scores positively correlated with activity in PCC.   Reward vs. punished errors P vs. ASPD showed decreased activity in right STG and MTG. P v. HC showed decreased activity in the right STG. |
| **88** | Jiang et al. (2016) | 2 ASPD v. HC | 27 ASPD (0) 20.30 25 HC 21.13 | Previous mental disorder or behavioral disorder caused by brain damage.  Covariates: age, IQ and education matched HC. | AB: youth offenders with ASPD; DSM-IV (I); PDQ (S) | 3T MRI Whole-brain VBA | ASPD vs. C showed reduced cortical thickness bilaterally in the SFG, rostral ACC, precuneus, OFC, insula, in the left STS, STG, and in the right MFG.  ASPD vs. C showed increased surface area bilaterally in STG, SFG, precuneus, in the left STS, mOFC, postcentral gyrus, in the right STG, MFG, insula, precentral gyrus, SMG, and parahippocampal gyrus. |
| **89** | Motzkin et al. (2011) | 4 P1 (32.2) v. C (14.6) P2 (31.9) v. C (14.2) | 14 P1 (0) 32.9 13 C1 (0) 31.7 20 P2 (0) 32.6 20 C2 (0) 31.1 | Age > 45, IQ < 70, history of psychosis, bipolar disorder, significant head injury or postconcussion symptoms, current psychotropic medication  Covariates: subjects divided into psychopaths (P) and nonpsycopaths (C); SUD; DTI and fMRI study subjects are denoted as 1 and 2, respectively. | AB: male offenders from a medium-security correctional facility  P: PCL-R (I) | 1.5T DTI + fMRI; rest ROI-A VBA, DT | DTI: P1 vs. C1 showed decreased FA in the whole brain compared to C. Relative to this reduction, FA in the right UF was also decreased in P1 vs. C1.   fMRI: P2 vs. C2 showed decreased connectivity between the right amygdala and anterior vmPFC, as well as between precuneus/PCC and vmPFC within the DMN. |
| **90** | Kumari et al. (2013) | 4 ASPD v. HC | 13 ASPD (0) 32.20 15 HC (0) 32.12 | All: age < 18 or > 55, current substance abuse, history of neurological conditions or head injury, non-native in English  HC: history of a mental disorder or violence  Covariates: age, gender, and IQ matched HC; psychosocial deprivation | AB: male ASPD inmates from medium and high security psychiatric hospitals; The Gunn and Robertson criminal profile (I). | 1.5T MRI Whole-brain and ROI VBA | Psychosocial deprivation in ASPD negatively correlated with the volume of the right thalamus compared to HC. |
| **91** | Jiang et al. (2017) | 2 ASPD v. HC | 20 ASPD (0) 21.8 23 HC (0) 22.1 | Previous mental disorder or behavioral disorder caused by brain damage.  Covariates: age, IQ and education matched HC | AB: youth offenders with ASPD; DSM-IV (I) and PDQ (S); BIS-11 (S); HBICA (S); | 3T DTI Whole-brain TBSS | ASPD vs. HC showed: decreased FA bilaterally in the IFOF, ACR, in the right UF, ALIC, middle frontal blade, in the left SLF, RPIC, SCR, fornix/stria terminalis, postcentral blade, and parieto-temporal blade; increased FA in the right CT, SLF, middle frontal blade, in the left IFOF and superior frontal blade; decreased AD bilaterally in SLF, CC, SCR, in the left PCR, ACR, ALIC, RPIC, PLIC, PTR including optic radiation, fornix/stria terminalis, postcentral blade, parieto-temporal blade, inferior frontal blade, in the right precentral blade and superior frontal blade; increased RD in the right SLF, superior frontal blade, in the left ACR, IFOF, ILF and temporal blade.   BIS score negatively correlated with AD in CC, left PCR, and left PTR. HBICA scores positively correlated with RD in the right SLF, and left IFOF. |
| **92** | Narayan et al. (2007) | 4 ASPD v. HC | 14 ASPD (0) 33.5 15 HC (0) 32.1 | All: age < 18 or > 55, Axis I disorders (except SUD)  HC: Axis I and II disorders  Covariates: SUD; age, gender and socioeconomic status matched HC | AB: violent offenders with ASPD from a high security hospital; DSM-IV (I) | 1.5T MRI ROI VBA | ASPD showed decreased GMV in medial inferior frontal cortices and right sensory-motor areas compared to HC. |
| **93** | Cohn et al. (2016) | 2 NC (24.3) | 134 (15) 17.7 | N/A  Covariates: subjects recruited from a longitudinal study; ADHD, DBD, PTSD; | AB: persons arrested before the age of 12; NIMH DISC-IV (I); RPQ (S); YSR (S); CBCL (S)  CU: YPI (S) | 3T MRI Whole-brain and ROI-A VBA | CU traits negatively correlated with GMC in right insula, and left amygdala. CD symptoms positively correlated with left amygdala GMC. No other associations found. |
| **94** | Murray, Shaw, Forbes, & Hyde (2017) | 2 NC | 144 S (0) 20 | N/A  Covariates: subjects recruited from a longitudinal study; DSM-IV Axis I disorders, IQ and ADHD | AB: ABQ (S); APSD (S); ASPD DSM-IV (I); criminal record transcripts;  CU: APSD (C) | 3T fMRI; task Whole-brain and ROI VBA | Reward anticipation: AB negatively correlated with activity in the right VS and left MFG (vlPFC). CU traits negatively correlated with activity in right vlPFC and MOG.   Loss anticipation: AB negatively correlated with activity in the left vlPFC and left IPL. No associations for CU traits. |
| **95** | Gregory et al. (2012) | 2 P (28.1) v. ASPD (16.4) v. HC (3.8) | 17 P (0) 31.8 27 ASPD (0) 36.1 22 HC (0) 32.4 | All: DSM-IV Axis I disorders, head injury resulting in loss of consciousness > 1h, significant visual och hearing impairments, reading age below 10 years, non-native in English  HC: criminal offense, ASPD, PCL-R > 20  Covariates: ASPD males divided to psychopaths (P) and nonpsychopaths (ASPD); IQ, age and SUD matched ASPD and P. | AB: male probationers with ASPD convicted for violent crimes; DSM-IV (I)  P: PCL -R (I) | 1.5T MRI ROI-A VBA | P vs. HC showed reduced GMV bilaterally in the MFG, SFG, IFG, insula, postcentral gyrus, in the right temporal pole and precentral gyrus. P vs. ASPD showed decreased GMV in the left ITG, bilateral MFG, and in the right temporal pole. No associations between ASPD and HC. |
| **96** | Larson et al. (2013) | 4 P (31.29) v. C (13.38) | 24 P (0) 33.13 25 C (0) 32.08 | Age < 18 or > 45, IQ < 70, diagnosis of psychosis, bipolar disorder, or schizophrenia; a history of neurological disorder or head injury; reading level below 4th grade, current psychotropic medication.  Covariates: subjects divided to psychopaths (P) and nonpsychopaths (C); IQ and age matched | AB: male offenders from a medium-security correctional facility   P: PCL-R (I) | 1.5T fMRI; task Whole-brain and ROI VBA | Differences only in early alternative attentional focus in instructed fear task: P vs. C showed decreased activity in the right amygdala; and increased activity in the left IFG (dlPFC), MFG (dlPFC) and SFG (mid-vlPFC). Right amygdala activity negatively correlated with activity in the left dlPFC and mid-vlPFC. |
| **97** | Freeman et al. (2015) | 4 P (31.34) v. C (20.08) | 22 P (0) 28.73 22 C (0) 34.77 | DSM-IV Axis I disorders, a history of psychosis in self or first degree relative, any event that resulted in loss of consciousness for more than 10 minutes.  Covariates: subjects divided to high psychopaths (P) and medium psychopaths (C); IQ, SUD matched; age | AB: male inmates; DSM-IV (I)  P: PCL-R (I) | 1.5T fMRI; task ROI VBA | Task vs. baseline in DMN: P showed no task-induced deactivation. C showed task-induced deactivation in posteromedial cortices, medial prefrontal cortices, and left lateral parietal cortex. F1 positively correlated with activity in posteromedial cortices. |
| **98** | Sitaram et al. (2014) | 2, 4 NC (12) | 4 S (0) 31.5 | N/A  Covariates: age | AB: persons on trial for or parolees convicted for sexual offenses  P: LP (S); PCL-SV (I) | 3T rtfMRI; task ROI VBA | One psychopathic criminal learned to regulate anterior insula by employing negative emotional imageries in conjunction with contingent feedback. |
| **99** | Marsh & Cardinale (2014) | 1 NC (304.5) | 33 S (39) 22 | N/A  Covariates: N/A | AB: RPQ (S)  P: PPI-R (S) | 3T fMRI; task Whole-brain and ROI VBA | Main effect of emotionally valenced written statements: in all subjects increased activity bilaterally in the precuneus and cuneus, in the left IFG, caudate, in the right MFG, CG, precentral gyrus and postcentral gyrus.   Fear was the only emotion category showing group differences. Psychopathy positively correlated with activity in the right MFG. Psychopathy negatively correlated with activity in right amygdala, parahippocampal gyrus, and bilateral precuneus. |
| **100** | Lindner et al. (2017) | 1, 2 NC (3.32) | 99 S (100) 24.23 | N/A  Covariates: SUD, age, IQ | AB: DSM-IV (I); MCVI (I)  P: PCL-SV (I) | 3T DTI Whole-brain and ROI-M TBSS PT | Facet 1 (interpersonal) scores negatively correlated with FA in bilateral UF; and AD in bilateral fornix. Facet 1 scores positively correlated with RD in the right UF. Facet 2 (affective) scores negatively correlated with FA adjacent to the left posterior fusiform gyrus; and AD adjacent to the left posterior ITG. Facet 3 (lifestyle) scores negatively correlated with FA adjacent to the left posterior fusiform gyrus. Facet 4 (antisocial) no correlations found. |
| **101** | Passamonti et al. (2012) | 2, 4 CD (2.6) v. HC (1.9) | 13 CD (0) 18.4 13 HC (0) 18.6 | All: IQ < 85, the presence of a pervasive developmental disorder or chronic physical illness  HC: IQ > 115  Covariates: age, gender, IQ, and socioeconomic status matched HC; ADHD | AB: persons from Pupil Referral Units and Youth Offending Services; K-SADS (I)  P: YPI (S) | 3T DTI Whole-brain and ROI-M VBA DT | CD showed increased FA, increased AD and decreased RD bilaterally in the external capsule and UF compared to HC. |
| **102** | Hoppenbrouwers et al. (2013) | 2, 3 P (28.1) v. HC | 11 P (0) 33.5 11 HC (0) 32.1 | A diagnosis of schizophrenia or any primary psychotic disorder, bipolar disorder, depressive or anxiety disorders and other personality disorders, current SUD, a history of traumatic brain injury, seizures, or stroke.  Covariates: age and gender matched HC | AB: parolees convicted for violent crimes  P: PCL-R (I) | 1.5T DTI Whole-brain TBSS PT | P showed WM deficits in amygdalo-prefrontal and striato-thalamo-frontal networks: reduced FA bilaterally in UF, IFOF, ATR and anterior CG. F1 negatively correlated with FA in the left UF, IFOF and ATR. F2 negatively correlated with FA in the right UF, IFOF and ATR. |
| **103** | Sundram et al. (2012) | 4 P (26) v. HC | 15 P (0) 39 15 HC (0) 37 | Comorbid psychiatric illness, SUD, neurological or extracerebral disorder affecting brain function.  Covariates: age, gender and IQ matched HC | AB: inmates with ASPD from specialist forensic units; ICD-10 (I)  P: PCL-R (I) | 1.5T DTI Whole-brain VBA | P showed reduced FA bilaterally in CC, IC and IFOF, in the right ACR, UF, in the left ILF and PTR compared to HC. P showed increased MD in the right IFOF, UF, CC and ACR compared to HC. Total PCL-R score and F2 negatively correlated with FA in the frontal lobe. F2 positively correlated with MD in frontal lobe. |
| **104** | Holz et al. (2017) | 2  NC | 178 S (58) 25 | Heart pacemaker, neurological abnormalities, history of seizures, unconsciousness or head trauma, current psychiatric disorders, and psychotropic medication.  Covariates: gender, obstetric adversity, lifetime substance abuse including nicotine dependence, alcohol abuse and lifetime cannabis abuse; parental ASPD diagnosis | AB: persons with prior CD diagnosis; DSM-IV (I); K-SADS (I); MPI (I); YASR (S)  CU: PSD (I) | 3T fMRI; task 3T MRI ROI VBM | Childhood family adversity increased the level of CD and ADHD diagnoses during childhood and adolescence, lifetime impulsivity and CU traits. Prior CD diagnoses were associated with increased impulsivity, higher aggression and CU traits as well as with decreased performance time in monetary trials. CD diagnoses were associated with decreased amygdalar activity in emotional face recognition task and decreased VS activity in reward-related task. |
| **105** | Howner et al. (2012) | 4 P (20) v. ASPD (11) v. HC (0.5) | 7 P (0) 27 7 ASPD (0) 30 12 HC (0) 28 | DSM-IV axis I diagnoses, difficulties in reading and understanding Swedish, an acute psychotic state at the time of assessment, and acute involuntary psychiatric treatment.  Covariates: age, educational level, occupation, IQ | AB: ASPD and psychopathic inmates on pretrial forensic evaluation and charged with mostly violent offences; DSM-IV (I)  P: PCL-SV (I) | 1.5T MRI ROI-A VBA | P vs. ASPD showed no differences in cortical thickness. P vs. HC showed cortical thinning in temporal lobes and the entire right hemisphere. ASPD vs. HC showed decreased global brain volume. Total PCL-SV score negatively correlated with GMV in bilateral temporal lobes and the right hemisphere. F1 negatively correlated with the GMV in bilateral temporal lobes. F2 correlated negatively with the GMV in the right frontal lobe, bilateral temporal lobes, the right parietal lobe and the right hemisphere. |
| **106** | Molenberghs et al. (2014) | 1 NC | 48 S (50) 22.5 | A completed psychology course.  Covariates: N/A | P: SRP-III (S) | 3T fMRI; task VBA | Rewarding others was associated with increased activity in the bilateral putamen and mOFC. Punishing other was associated with increased activity in the mPFC, dACC, bilateral OFC, bilateral anterior insula and right pSTS. In the latter setting, psychopathy scores positively correlated with activity in the mPFC, dACC, left IFG/anterior insula, right IFG, and right pSTS. |
| **107** | Volman et al. (2016) | 4 P (30.4) v. HC | 15 P (0) 37.8 19 HC (0) 40.7 | DSM-IV axis I and II disorders (apart from ASPD), alcohol use exceeding 3 units/day, cannabis, or other illicit drug use 1 week before, psychotropic medication other than oxazepam 5 d before, 1 unit of alcohol or oxazepam use within 24 h before the experiment; history of trauma capitis; visual and auditive disorder; and neurological disorder.  Covariates: age and IQ matched HC | AB: psychopathic offenders from psychiatric institutes  P: PCL-R | 1.5T fMRI; task ROI-A VBA | In a facial recognition task, P vs. HC showed decreased activity in the anterior PFC as well as decreased anterior PFC–amygdala connectivity. P with relatively lower testosterone levels showed a neural activity and connectivity pattern resembling that of HC, while this pattern was absent in those with higher testosterone levels. |
| **108** | Decety et al. (2015) | 4 P (≥30) v. MP (21-29) v. HC ≤20) | 38 P (0) 32.4 67 MP (0) 33.1 50 C (0) 31.6 | Age <18 or > 55 years, nonfluency in English, reading level lower than 4th grade, IQ score lower than 80, a history of seizures, prior head injury with loss of consciousness > 30 min, DSM-IV axis I diagnosis, a lifetime history of a psychotic disorder in self or in a first degree relative, current alcohol or drug use  Covariates: subjects divided into groups of high (P), medium (MP) and low (C) psychopathy; age and IQ matched | AB: male offenders from a medium security correctional facility  P: PCL-R (I) | 1.5T fMRI; task | In a task involving recognition harmful vs. helpful scenarios, P vs. C showed decreased activity in the right pSTS/TPJ, dmPFC, dlPFC, temporal pole, and ACC. These areas negatively correlated with F1. Moreover, F1 positively correlated with activity in the VS when viewing harmful interactions. During emotional evaluation of the setting, P vs. C showed increased activity in these areas. In addition, when evaluating harmful consequences for the victim, high PCL-R scorers showed an increase in activity bilaterally in the pSTS/TPJ, hippocampus, amygdala, and ACC. In fact, a higher PCL-R score was associated with a greater accuracy rate in identifying the emotions of the victim of a harmful action or the recipient of a helpful interaction. |
| **109** | Raine et al. (2011) | 1 ASPD v. HC v. C | 18 ASPD (0) 32.9 30 HC (0) 31.3 24 C (0) 30.2 18 PC (0) 12 FASPD (100) 33.9 | Age < 21 or > 45, non-fluency in English, history of epilepsy, claustrophobia, pacemaker and metal implants.  Covariates: DSM-IV axis I and II disorders; PC = ASPD individuals without DSM-IV axis I and II comorbidities; C = substance-dependent control group without ASPD; FASPD = female ASPD group | AB: Self-Report Crime Checklist (S); DSM-IV (I) | 1.5T MRI Whole-brain and ROI VBA | ASPD vs. male controls showed a 9% reduction in GMV in the OFC, a 17% reduction in the mPFC, and a 16% reduction in the right rectal gyrus. Decreased GMV in the mPFC and OFC were associated with increased ASPD symptoms and criminal offending in both genders. ASPD males vs. females showed reduced GMV in the OFC and mPFC. |
| **110** | Beckwith et al. (2018) | 1 NC | 155 S (58) 26 | N/A  Covariates: age, IQ, the use of two different 3T scanners | P: PPI (S) | 3T MRI Whole-brain VBA | Total psychopathy score negatively correlated with WMV in the cerebellum and brain stem in the whole group. No associations for GMV. Females vs. males showed increased GMV in the frontal, temporal, parietal and occipital cortices, which positively correlated with total psychopathy score. Females did not display changes in the WM. Males vs. females showed decreased GMV and WMV in the prefrontal cortex and cerebellum. Decreased GMV and WMV was associated with increased blood lead levels in the bilateral frontal and right temporal cortices. Childhood lead exposure may negatively affect GMV and WMV and, thus, be attributable to increased display of psychopathic traits. |
| **111** | Reniers et al. (2012) | 1 NC | 24 S (0) 22 | Self-reported treatment for any psychiatric illness within the last year, current alcohol abuse or dependence (use of more than 20 units per week), history of serious head injury (more than 5 min loss of consciousness or overnight hospital stay), serious medical or neurological conditions  Covariates: N/A | P: LP (S) | 3T fMRI; task ROI VBA | Moral vs. non-moral decision making was associated with increased activity in the mPFC, dlPFC, IPL, precuneus and SMG in the whole group. Primary psychopathy was associated with increased activity in the dlPFC and mPFC during moral decision making, suggesting that moral decision making involves a greater degree of introspective abilities. |
| **112** | Kumari et al. (2006) | 4 ASPD v. HC | 10 ASPD (0) 31.30 13 HC (0) 33.31 | N/A  Covariates: SUD | AB: DSM-IV (I) | 1.5T fMRI ROI VBA | In an n-back task, ASPD was associated with slightly decreased working memory performance compared to HC. Further, ASPD vs. HC showed diminished activity in the left frontal gyrus, ACC, and precuneus. |
| **113** | Barkataki et al. (2008) | 4 ASPD v. HC | 14 ASPD (0) 33.5 14 HC (0) 32.1 | Age < 18 or > 45 years, non-native in English, current substance abuse, neurological conditions or head injuries, axis I disorder diagnosis.  Covariates: N/A | AB: The Gunn and Robertson Violence Scale (I); DSM-IV (I) | fMRI  N/A | ASPD group did not display decreased activity in frontal lobes or ACC during NoGo conditions. ASPD did show decreased activity in the thalamus during this condition. This may contribute to impaired control inhibition in ASPD. |
| **114** | Kumari et al. (2009) | 4 ASPD v. HC | 13 ASPD (0) 32.85 14 HC (0) 33.14 | Age < 18 or > 55, substance abuse, neurological conditions, non-native in English  Covariates: age and IQ matched HC | AB: ASPD inmates; DSM-IV (I); The Gunn and Robertson Violence Scale (I) | 1.5T fMRI VBA | ASPD showed diminished striatothalamic activity during exposure to visual threat cues. They also showed aberrant activity in temporal and occipital regions compared to HC. |
| **115** | Buckholtz et al. (2010) | 1 NC | 24 S (67) 18-35 | A history of substance abuse, current tobacco use, alcohol intake greater than 8 ounces of whiskey or equivalent per week, use of psychostimulants (excluding caffeine) more than twice in the subject’s lifetime or at all in past 6 months, any psychotropic medication for the past 6 months other than occasional use of benzodiazepines for sleep, history of psychiatric illness, significant medical condition, any condition which would interfere with MRI or PET studies.  Covariates: gender | P: PPI (S) | 3T fMRI; task ROI VBA | In a reward anticipation setting, impulsive antisociality (PPI-IA) factor scores positively correlated with BOLD signal in the right NAcc. |
| **116** | Bobes et al. (2013) | 1 V (10.6) v. NV (3.0) | 25 V (0) 30.63 29 NV (0) 28.93 | Inability to see small objects without the aid of spectacles, chronic disease.  Covariates: age, cultural level, and socioeconomic status-matched groups. | AB: Buss–Durkee Hostility Inventory (S); Plutchik Impulsivity Scale (S); Novaco Anger Scale (S); RPQ (S);  P: PCL-R (I); LP (S) | 1.5T fMRI; task ROI-A VBA | V vs. NV showed a smaller difference between responses to fearful and neutral facial expressions (F/N). F/N positively correlated with GMC in the left amygdala. No correlations found for CU traits. The authors argument that amygdalar hyper-reactivity to social signals may be characteristic of reactive aggression. |
| **117** | Kolla et al. (2014) | 1, 3 P (27.7) v. ASPD (15.2) v. HC (3.8) | 9 P (0) 38.7 15 ASPD (0) 35.0 13 HC (0) 35.1 | N/A  Covariates: age, IQ and SUD matched ASPD groups. | AB: violent male probationers with ASPD; Early Trauma Inventory (S)  P: PCL-R (I) | 1.5T MRI Whole-brain VBA | P vs. ASPD showed decreased GMV in bilateral temporal poles, right uncus, and right posterior cerebellum and reported more physical abuse in childhood. |
| **118** | Kolla et al. (2016) | 1, 3 NC | 19 ASPD (0) 36.0 | A history of psychotic, major depressive, or bipolar syndrome; current non-alcohol substance abuse or dependence; current psychotropic medication or cigarette smoking.  Covariates: IQ | AB: violent offenders with ASPD from community and probation services; DSM-IV (I); BIS-11 (S); NEO-PI-R (S)  P: PCL-R (I) | 3T fMRI; rest ROI VBA | MAO-A levels. positively correlated with functional coupling between VS and dmPFC; and negatively between VS and right hippocampus. VS-hippocampus connectivity negatively correlated with impulsivity measures suggesting that MAO-A levels in the striatum affect impulsivity in ASPD. |

Notes: The Type and Design column includes mean psychopathy score in parentheses. The Sample column includes number of subjects, followed by percentage of females in parentheses, followed by mean age as reported.

Type of sample**:** 1 = community; 2 = high-risk; 3 = clinic; 4 = forensic;

Design acronyms: NC = non-categorical; P = psychopath or high psychopath group; MP = intermediate psychopath group; C = control group; HC = healthy control group; ASPD = antisocial personality disorder group; V = violent groups; NV = non-violent group; CD = conduct disorder group; ACD = adolescence on-set CD group; ECD = childhood on-set CD group; SP = successful psychopath group; UP = unsuccessful psychopath group;

MRI and fMRI acronyms: ACC = anterior cingulate cortex; AIC = anterior insular cortex; AG = angular gyrus; BLA = basolateral complex of amygdala; CB = cerebellum; CE central subnucleus of amygdala; CG= cingulate gyrus; dACC = dorsal anterior cingulate cortex; DMN = default mode network; dmPFC = dorsomedial prefrontal cortex; dlPFC = dorsolateral prefrontal cortex; FPC = frontopolar cortex; FPN = frontoparietal network; GP = globus pallidus; IFG = inferior frontal gyrus; ITG = inferior temporal gyrus; IOG = inferior occipital gyrus; IPL= inferior parietal lobule; MCC = midcingulate cortex; MFC = medial frontal cortex; MFG = middle frontal gyrus; MOG middle occipital gyrus; mOFC = medial orbitofrontal cortex; MTG = middle temporal gyrus; NAcc = nucleus accumbens; OFC = orbitofrontal cortex; PAG = periaqueductal gray matter; PCC = posterior cingulate cortex; PFC = prefrontal cortex; pSTS = posterior superior temporal sulcus; RG = rectal gyrus; SFG= superior frontal gyrus; SMA = supplementary motor area; SMG = supramarginal gyrus; SN = salience network; SPL = superior parietal lobule; STC = superior temporal cortex; STG = superior temporal gyrus; TPJ = temporoparietal junction; vmPFC = ventromedial prefrontal cortex; vlPFC = ventrolateral prefrontal cortex

DTI acronyms: ACR =anterior corona radiata; AD = axial diffusivity; ALIC = anterior limb of internal capsule; ATR = anterior thalamic radiation; CC = corpus callosum; CG = cingulum/cingulate gyrus; CT = corticospinal tract; Fmaj = forceps major; Fmin = forceps minor; FA = fractional anisotropy; IC = internal capsule; IFOF = inferior fronto-occipital fasciculus; ILF = inferior longitudinal fasciculus; MD = mean diffusivity; PCR = posterior corona radiata; PLIC = posterior limb of internal capsule; PTR = posterior thalamic radiation; RD = radial diffusivity; RPIC = retrolenticular part of internal capsule; SCR = superior corona radiata; SLF = superior longitudinal fasciculus; UF = uncinate fasciculus

Behavior acronyms: AB = antisocial behavior; ABQ = Antisocial Behavior Questionnaire; ADHD = attention deficit hyperactivity disorder AQ-SF = Aggression Questionnaire, Short Form; ASI = Addiction Severity Index; ASPD = antisocial personality disorder ASR = Adult Self-Report; ASRS = Adult Self-Report Scale for ADHD; BIS-11 = Barratt Impulsiveness Scale 11; BPAQ = Buss Perry Aggression Questionnaire; C = calculated composite score; CBCL = Child Behavior Checklist; CD = conduct disorder; CPS = Child Psychopathy Scale, Revised; CU = callous-unemotional; DBD = disruptive behavioral disorder; DSM-IV = semi-structured diagnostic interview for DSM-IV personality disorders; HBICA = Health-Risk Behavior Inventory for Chinese Adolescents; ICD-10 = semi-structured diagnostic interview for ICD1-0 personality disorders; ICU = inventory of callous-unemotional traits; IM-P = Interpersonal Measure of Psychopathy; IPAS = Impulsive/Premeditated Aggression Scale; IRI = Interpersonal Reactivity Index; K-SADS = Schedule for Affective Disorders and Schizophrenia for School‐Age Children; LP = Levenson Primary and Secondary Psychopathy Scales; MCVI = MacArthur Community Violence Instrument; MPQ-BF = Multidimensional Personality Questionnaire, Brief Form; NEO-FFI = NEO Five Factor Inventory; NEO-PI-R = NEO Personality Inventory Revised; NYS-AD= National Youth Survey Adult Version; PCL-R = Psychopathy Checklist Revised; PCL-SV = Psychopathy Checklist Screening Version; PCL-YV = Psychopathy Checklist Youth Version; PDQ = Personality Diagnostic Questionnaire 4+; PPI-R = Psychopathic Personality Inventory Revised; PPI-S = Psychopathic Personality Inventory, Short Version; RPQ = Reactive-Proactive Aggression Questionnaire; SRD = Self-Report for Delinquency; SRP-III = Self-Report of Psychopathy III; SRP-SF = Self-Report Psychopathy Scale Short-Form; STAXI = State-Trait Expression Inventory; SUD = substance use disorder; TRF = Teacher Report Form; TRiPM = Triarchic Psychopathy Measure; VHQ = Violence History Questionnaire; VRAG = Violence Risk Appraisal Guide; YPI = youth psychopathic traits inventory; YPI-S = youth psychopathic traits inventory, short version; YSR = Youth Self-Report; I = structured interview; P = psychopathy; S = self-reported; P = parent-reported; T = teacher-reported

Supplementary Table 2: List of excluded records with reasons

|  | Author and year | Reason for exclusion |
| --- | --- | --- |
| 1 | Sterzer, Stadler, Poustka, & Kleinschmidt (2007) | Mean age < 17.50 yrs |
| 2 | Sarkar et al. (2016) | Mean age < 17.50 yrs |
| 3 | Sterzer, Stadler, Krebs, Kleinschmidt & Poustka (2005) | Mean age < 17.50 yrs |
| 4 | Pu, Luo, Jiang, Gao, Ming, & Yao (2017) | Mean age < 17.50 yrs |
| 5 | Zhou, Yao, Fairchild, Zhang, & Wang (2015) | Mean age < 17.50 yrs |
| 6 | Wu, Zhang, Dong, Wang, & Yao (2017) | Mean age < 17.50 yrs |
| 7 | Puzzo et al. (2018) | Mean age < 17.50 yrs |
| 8 | Fairchild et al. (2014) | Mean age < 17.50 yrs |
| 9 | Byrd, Hawes, Burke, Loeber, & Pardini (2018) | Mean age < 17.50 yrs |
| 10 | Michalska, Zeffiro, & Decety (2016) | Mean age < 17.50 yrs |
| 11 | Fairchild et al. (2013) | Mean age < 17.50 yrs |
| 12 | Raschle et al. (2018) | Mean age < 17.50 yrs |
| 13 | Breeden, Cardinale, Lozier, Van Meter, & Marsh (2015) | Mean age < 17.50 yrs |
| 14 | Wallace et al. (2014) | Mean age < 17.50 yrs |
| 15 | Klapwijk et al. (2016a) | Mean age < 17.50 yrs |
| 16 | Aghajani et al. (2017) | Mean age < 17.50 yrs |
| 17 | Zhou et al. (2016) | Mean age < 17.50 yrs |
| 18 | Jiang et al. (2016) | Mean age < 17.50 yrs |
| 19 | Aghajani et al. (2016) | Mean age < 17.50 yrs |
| 20 | Rubia et al. (2008) | Mean age < 17.50 yrs |
| 21 | Zhang et al. (2018) | Mean age < 17.50 yrs |
| 22 | Haney-Caron, Caprihan, & Stevens (2014) | Mean age < 17.50 yrs |
| 23 | Marsh et al. (2013) | Mean age < 17.50 yrs |
| 24 | Klapwijk et al. (2016b) | Mean age < 17.50 yrs |
| 25 | Dalwani et al. (2015) | Mean age < 17.50 yrs |
| 26 | Yang et al. (2015a) | Mean age < 17.50 yrs |
| 27 | Sarkar et al. (2013) | Mean age < 17.50 yrs |
| 28 | Zhang et al., 2015a | Mean age < 17.50 yrs |
| 29 | Thijssen & Kiehl (2017) | Mean age < 17.50 yrs |
| 30 | Rijsdijsk et al. (2010) | Mean age < 17.50 yrs |
| 31 | Sakai et al. (2017) | Mean age < 17.50 yrs |
| 32 | Zhang et al. (2015b) | Mean age < 17.50 yrs |
| 33 | Finger et al. (2012) | Mean age < 17.50 yrs |
| 34 | Zhang et al. (2014) | Mean age < 17.50 yrs |
| 35 | Zhang et al. (2017) | Mean age < 17.50 yrs |
| 36 | Walters & Kiehl (2015) | Mean age < 17.50 yrs |
| 37 | Steele, Rao, Calhoun & Kiehl (2017) | Mean age < 17.50 yrs |
| 38 | Lozier, Cardinale, Van Meter, & Marsh (2014) | Mean age < 17.50 yrs |
| 39 | Menks et al. (2017) | Mean age < 17.50 yrs |
| 40 | Huebner et al. (2008) | Mean age < 17.50 yrs |
| 41 | Harenski, Harenski, & Kiehl (2014) | Mean age < 17.50 yrs |
| 42 | Sebastian et al. (2012) | Mean age < 17.50 yrs |
| 43 | Sebastian et al. (2014) | Mean age < 17.50 yrs |
| 44 | De Brito (2010) | Mean age < 17.50 yrs |
| 45 | White et al. (2012a) | Mean age < 17.50 yrs |
| 46 | White et al. (2012b) | Mean age < 17.50 yrs |
| 47 | Marsh et al. (2008) | Mean age < 17.50 yrs |
| 48 | Marsh et al. (2011) | Mean age < 17.50 yrs |
| 49 | Sarkar et al. (2015) | Mean age < 17.50 yrs |
| 50 | Broulidakis et al. (2016) | Mean age < 17.50 yrs |
| 51 | De Brito (2009) | Mean age < 17.50 yrs |
| 52 | De Brito et al. (2011) | Mean age < 17.50 yrs |
| 53 | Yang et al. (2015b) | Mean age < 17.50 yrs |
| 54 | Fowler & Blair (2011) | Mean age < 17.50 yrs |
| 55 | Viding et al. (2012) | Mean age < 17.50 yrs |
| 56 | Finger et al. (2011) | Mean age < 17.50 yrs |
| 57 | Lu, Zhou, Zhang, Wang, & Yuan (2017) | Mean age < 17.50 yrs |
| 58 | Cohn et al. (2013) | Mean age < 17.50 yrs |
| 59 | Cohn et al. (2016) | Mean age < 17.50 yrs |
| 60 | Cohn et al. (2015) | Mean age < 17.50 yrs |
| 61 | Schwenk et al. (2017) | Mean age < 17.50 yrs |
| 62 | Glenn, Raine, & Schug (2009) | Letter to the Editor |
| 63 | Veroude et al. (2016) | ADHD as inclusion criterion |
| 64 | Calzada-Reyes et al. (2015) | Book chapter |
| 65 | Yang et al. (2012) | Demographic data missing |
| 66 | Decety, Michalska, Akitsuki, & Lahey (2009) | ADHD as inclusion criterion |
| 67 | Yang, Raine, Colletti, Toga, & Narr (2009 | Letter to the Editor |
| 68 | Aoki, Inokuchi, Nakao, & Yamasue (2014) | Meta-analysis |
| 69 | Da Cunha-Bang et al. (2015) | Conference proceedings |
| 70 | Sun, Yu, Yuan, Gao, & Yao (2016) | Chinese |
| 71 | Jiang et al. (2015) | Chinese |
| 72 | Blair (2012) | Not a neuroimaging study |
| 73 | Yildirim & Tureli (2015) | Turkish |
| 74 | Sobhani (2014) | Dissertation |
| 75 | Yang (2009) | Dissertation |
| 76 | Blair (2009) | Comment |
| 77 | Stadler (2011) | Conference proceedings |
| 78 | Fowler, Schechter, Pine, Sinclair, & Blair (2009) | Full record not found |
| 79 | Pape et al. (2013) | Conference proceedings |
| 80 | Gonzalez-Madruga et al. (2019) | Full record not found (in print) |
| 81 | Porges & Decety (2011) | Conference proceedings |
| 82 | Cohn et al. (2013) | Conference proceedings |

Supplementary Table 3: Grouping of functional studies

| Setting / Area of Interest | Record |
| --- | --- |
| Fair vs. unfair | 3, 32, 67 |
| Moral issue | 7, 18, 33, 56, 64, 70, 82, 99, 108, 111 |
| Viewing emotional content including facial expressions | 6, 14, 15, 28, 30, 34, 35, 36, 39, 47, 55, 57, 58, 65, 71, 72, 81, 96, 98, 104, 107, 116 |
| Reward | 31, 45, 63, 66, 76, 83, 87, 106, 115 |
| DMN | 10, 26, 27, 40, 46, 50, 54, 79, 89, 97 |
| Lying / Deception | 8, 9, 77 |

Supplementary Table 4: Prefrontal correlations with interpersonal-affective dimensions (complimentary for Table 2)

| No. | Record | Method | OFC | vmPFC | vlPFC | dmPFC | dlPFC | mPFC | FPC | LFC | PFgyri |
| --- | --- | --- | --- | --- | --- | --- | --- | --- | --- | --- | --- |
| 5 | Yoder et al. (2015a) | F |  |  |  |  |  |  |  |  |  |
| 6 | Decety el al. (2013) | F |  |  |  | a+/- |  |  |  |  |  |
| 7 | Seara-Cardoso et al. (2016) | F |  |  |  |  |  |  |  |  |  |
| 9 | Glenn et al. (2017) | F | a- |  |  |  | a- |  |  | a+ |  |
| 10 | Lindner et al. (2017) |  |  |  |  |  |  |  |  |  |  |
| 12 | Leutgeb et al. (2015) | S |  |  |  |  | g- |  |  |  |  |
| 14 | Decety el al. (2013) | F |  | a- |  | a+/- |  |  |  |  | a+/- |
| 16 | Fairchild et al. (2011) | S |  |  |  |  |  |  |  |  |  |
| 27 | Cohn et al. (2015a) | F |  |  |  |  |  |  | c+ |  |  |
| 28 | Anderson et al. (2017) | F |  | a+ |  |  |  |  |  |  |  |
| 30 | Contreras-Rodríguez et al. (2014) | F |  |  |  |  |  | a+ |  |  | a+ |
| 31 | Hosking et al. (2017) | F |  | c- |  |  |  |  |  |  |  |
| 35 | Vieira et al. (2017) | F |  |  |  |  |  |  |  |  |  |
| 37 | Sethi et al. (2015) | DTI |  |  |  |  |  |  |  |  |  |
| 38 | Cope et al. (2012) | S |  |  |  |  |  |  |  |  | g+ |
| 40 | Contreras-Rodríguez et al. (2015) | S |  |  |  | g- |  |  |  | g- |  |
| 40 | Contreras-Rodríguez et al. (2015) | F |  |  |  |  |  | c+ |  | c+ |  |
| 45 | Korponay et al. (2017a) | S |  |  |  |  |  |  |  |  |  |
| 48 | Glenn et al. (2010) | S |  |  |  |  |  |  |  |  |  |
| 49 | Wolf et al. (2015) | DTI |  |  |  |  |  |  |  |  |  |
| 52 | Baskin-Sommers et al. (2016) | S |  |  |  |  |  |  |  |  |  |
| 53 | Miskovich et al. (2018) | S |  |  |  |  |  |  |  |  |  |
| 54 | Philippi et al. (2015) | F |  |  |  |  |  |  |  |  | c- |
| 56 | Harenski et al. (2010) | F |  |  |  |  |  |  |  |  |  |
| 59 | Yang et all. (2009) | S |  |  |  |  |  |  |  |  |  |
| 60 | Pardini et al. (2014) | S |  |  |  |  |  |  |  |  |  |
| 64 | Harenski et al. (2014) | F |  |  |  |  |  |  |  |  |  |
| 65 | Zijlmans et al. (2018) | F |  | a- |  |  |  |  |  |  | a- |
| 69 | Schiffer et al. (2014) | F |  |  |  |  |  |  |  |  |  |
| 70 | Yoder et al. (2015b) | F |  |  |  |  | a- |  |  |  | a+/- |
| 71 | Decety el al. (2014) | F | a- | a- |  | a- |  |  |  |  | a+/- |
| 72 | Seara-Cardoso et al. (2015) | F |  |  |  |  |  |  |  |  | a- |
| 77 | Fullam et al. (2009) | F |  |  |  |  |  |  |  |  |  |
| 78 | Vieira et al. (2015) | S | g+ |  |  |  |  |  |  |  |  |
| 79 | Anderson et al. (2018) | F |  |  |  |  |  | a- |  |  |  |
| 80 | Pape et al. (2015) | DTI |  |  |  |  |  |  |  |  |  |
| 83 | Cope et al. (2014) | F |  |  |  |  |  |  |  |  | a+ |
| 85 | de Oliveira-Souza et al. (2008) | S | g- |  |  |  |  |  | g- |  |  |
| 86 | Lam et al. (2017) | S | g- |  |  |  |  |  |  |  |  |
| 93 | Cohn et al. (2016) | S |  |  |  |  |  |  |  |  |  |
| 94 | Murray et al. (2017) | F |  |  | a- |  |  |  |  |  |  |
| 97 | Freeman et al. (2015) | F |  |  |  |  |  |  |  |  |  |
| 102 | Hoppenbrouwers et al. (2013) | DTI |  |  |  |  |  |  |  |  |  |

Abbreviations: OFC = orbitofrontal cortex; vmPFC = ventromedial prefrontal cortex; vlPFC = ventrolateral prefrontal cortex; dmPFC = dorsomedial prefrontal cortex; dlPFC = dorsolateral prefrontal cortex; mPFC = medial prefrontal cortex; FPC = frontopolar cortex; LFC = lateral frontal cortex; up-and down-arrow denote increase or decrease, respectively; c = functional connectivity; a = activity; g = gray matter volume; w = white matter volume; plus and minus signs denote direction of relationship with F1 psychopathy.

Supplementary Table 5: Brain regions in psychopathy with GMV reductions unless otherwise specified in Comments

| Brain region | Record | Comments |
| --- | --- | --- |
| dmPFC | 12, 17 |  |
| FPC | 13, 22, 85 |  |
| OFC | 13, 22, 51, 62, 75, 85 |  |
| Frontal gyri | 22, 24, 41, 75, 95 |  |
| SMA | 22 |  |
| vmPFC | 40 |  |
| Ventral & lateral | 40 |  |
| dlPFC | 75 |  |
| Precentral gyri | 24, 95 |  |
| STG | 24, 41 |  |
| MTG | 13, 40 |  |
| STS | 43, 85 |  |
| Fusiform gyrus | 22, 40 |  |
| Temporal pole | 24, 51, 95 |  |
| Temporal cortex | 62, 105 | [105] found cortical thinning in the entire right hemisphere |
| Inferior temporal cortex | 51 |  |
| Postcentral gyri | 13, 17, 95 | [13] found increased WMV in parietal cortices |
| Precuneus | 17, 40, 22 |  |
| Occipital cortex | 17 | [13] found increased WMV in occipital cortices |
| Cuneus | 22 |  |
| Lateral | 24 |  |
| Peristriate cortex | 43 |  |
| Amygdala | 40, 51, 59, 60, 62, 78 | [22] found increased GMV |
| Hippocampus | 1, 40, 51 | [1] and [42] found abnormal morphology |
| Parahippocampal gyrus | 13, 51 |  |
| ACC | 22, 24 | [73] found no differences in ACC GMV |
| MCC | 41 | [53] found abnormal gyrification |
| PCC | 13, 17, 40, 43, 51 |  |
| Insula | 13, 15, 24, 40, 85, 95 |  |
| Putamen | 45, 48 | 78 found decreased GMV; 11 found normal GMV |
| Caudate | 12, 48, 78 | 11 found normal GMV |
| Globus Pallidus | 12, 48 |  |
| Nucleus Accumbens | 45 | 11 found decreased GMV |
| Cerebellum | 117 | [12] and [13] increased GMV; [13] found increased WMV; [110] found decreased WMV |

# Search Strings

Below are the search strings used for the systematic review. The search was conducted on the 4^th^ of February 2019.

**Pubmed**

((Psychopathy) OR (psychopathic) OR (callous unemotional) OR (antisocial personality disorder [mh]) OR (conduct disorder [mh]))

AND

((neuroimaging [mh]) AND (magnetic resonance imaging [mh]) OR (diffusion tensor imaging [mh]) OR (voxel-based morphometry) OR (vbm) OR (white matter [mh]) OR (gray matter [mh]) OR (connectivity))

252 results

**Medline (Ovid)**

1 psychopathy.mp. (2932)

2 exp Conduct Disorder/ (3150)

3 (callous adj unemotional).mp. [mp=title, abstract, original title, name of

substance word, subject heading word, floating sub-heading word, keyword heading

word, organism supplementary concept word, protocol supplementary concept word,

rare disease supplementary concept word, unique identifier, synonyms] (664)

4 psychopathic.mp. (2469)

5 White Matter/ (5199)

6 Gray Matter/ (2786)

7 Magnetic Resonance Imaging/ (367742)

8 voxel-based morphometry.mp. (4789)

9 functional magnetic resonance imaging.mp. (27805)

10 connectivity.mp. (48302)

11 exp Diffusion Tensor Imaging/ (7897)

12 exp Neuroimaging/ (157990)

13 exp Antisocial Personality Disorder/ (9135)

14 1 or 2 or 3 or 4 or 13 (13746)

15 5 or 6 or 7 or 8 or 9 or 10 or 11 (418302)

16 12 and 15 (56952)

17 14 and 16 (168)

168 results

**Embase**

('psychopathy'/exp OR 'antisocial personality disorder'/exp OR 'conduct disorder'/exp OR 'callous unemotional trait'/exp) AND ('neuroimaging'/exp AND ('nuclear magnetic resonance imaging'/exp OR 'functional magnetic resonance imaging'/exp OR 'diffusion tensor imaging'/exp OR 'voxel based morphometry'/exp OR 'white matter'/exp OR 'gray matter'/exp OR 'connectivity'/exp))

245 results

**PsycINFO and PsycARTICLES (Ovid)**

1 psychopathy.mp. (9767)

2 exp Conduct Disorder/ (4210)

3 (callous adj unemotional).mp. [mp=ti, ab, hw, tc, id, ot, tm, tx, ct] (1253)

4 psychopathic.mp. (8217)

5 White Matter/ (7780)

6 Gray Matter/ (4112)

7 Magnetic Resonance Imaging/ (18631)

8 voxel-based morphometry.mp. (3258)

9 functional magnetic resonance imaging.mp. (31779)

10 connectivity.mp. (24156)

11 exp Diffusion Tensor Imaging/ (2275)

12 exp Neuroimaging/ (84633)

13 exp Antisocial Personality Disorder/ (4523)

14 1 or 2 or 3 or 13 (16528)

15 5 or 6 or 7 or 8 or 9 or 10 or 11 (71837)

16 12 and 15 (42941)

17 14 and 16 (160)

160 results

**Criminal Justice Abstracts (EBSCO)**

((Psychopathy) OR (psychopathic) OR (antisocial personality) OR (conduct disorder))

AND (neuroimaging) AND ((diffusion tensor imaging) OR (dti) OR (voxel-based morphometry) OR (vbm) OR (“magnetic resonance imaging”) OR (mri) OR (“functional magnetic resonance imaging”) OR (fmri) OR (“white matter”) OR (“gray matter”) OR (connectivity))

13 results

**TOTAL 838 RESULTS**

1. Monika Sommer, 21^st^ February 2019, personal communication [↑](#footnote-ref-1)
